# Treatment characteristics and safety profiles of Belbuca®, buprenorphine patch, and oral schedule II opioids among chronic low back pain patients without a positive history of opioid-use disorder: a retrospective US commercial claims analysis

**DOI:** 10.3389/fpain.2026.1764842

**Published:** 2026-06-30

**Authors:** Vladimir Zah, Dimitrije Grbic, Filip Stanicic

**Affiliations:** Health Economics and Outcomes Research Department, ZRx Outcomes Research Inc., Mississauga, ON, Canada

**Keywords:** adverse events, buprenorphine buccal film, buprenorphine transdermal patch, chronic low back pain, insurance claims, opioid use disorder, real-world evidence, schedule II opioids

## Abstract

**Introduction:**

The objective of this retrospective study was to evaluate and compare the safety characteristics of patients with chronic low back pain (cLBP) without a recent positive history of opioid use disorder (OUD).

**Methods:**

This study was conducted using the Merative MarketScan® database (January 2019–December 2023). The first date of Belbuca®, buprenorphine patch, or oral schedule II (CII) opioid prescription was designated as the index date. The observational period covered a 6-month preindex period and a follow-up period that lasted until the end of index treatment or continuous healthcare coverage. Patients were required to have two low back pain diagnoses and no OUD in the preindex period and continuous healthcare coverage during the observational period. The primary outcomes were serious treatment-emergent adverse event (TEAE) rates reported as incidence rate ratios (IRR) or absolute incidence rate difference (IRD) per 1,000 person-years for TEAEs occurring in one cohort. Propensity-score matching was employed to balance differences in patient characteristics and minimize their impact on study outcomes.

**Results:**

There were no serious TEAEs associated with higher occurrence in the Belbuca® cohort compared with oral CII opioids. Belbuca® treatment was associated with a significantly lower rate of serious opioid abuse/dependence (IRD −33.76 per 1,000 person-years, *p* = 0.032), osteoarthritis (IRD −78.77 per 1,000 person-years, *p* = 0.001), urinary discomfort (IRD −146.28 per 1,000 person-years, *p* < 0.001), seizures (IRR 0.11, *p* = 0.019), dehydration (IRR 0.13, *p* = 0.003), abdominal pain (IRR 0.25, *p* < 0.001), and nausea/vomiting (IRR 0.30, *p* = 0.001). The subanalysis compared incidence rates of serious TEAEs between Belbuca® and buprenorphine patch cohorts. Belbuca® demonstrated higher rates of serious coronary artery disease (IRD 39.01 per 1,000 person-years, *p* = 0.035), cholecystitis (IRD 39.01 per 1,000 person-years, *p* = 0.035), and headache (IRD 39.01 per 1,000 person-years, *p* = 0.035). However, the buprenorphine patch cohort had higher incidence rates of serious QT prolongation (IRD −52.78 per 1,000 person-years, *p* = 0.009), opioid abuse/dependence (IRD −184.75 per 1,000 person-years, *p* < 0.001), confusion (IRR 0.10, *p* = 0.007), hypertension (IRR 0.22, *p* = 0.043), and cellulitis (IRR 0.41, *p* = 0.011).

**Conclusion:**

The study findings suggest that Belbuca® may have a favorable safety profile relative to oral CII opioids and buprenorphine patch treatments in cLBP patients without a positive history of OUD.

## Introduction

Chronic low back pain (cLBP) is one of the most common diagnoses and pain conditions in the US population, affecting ∼40% of adults during their lifetime (1). Approximately 6% of all emergency department (ED) visits are attributed to a cLBP diagnosis. This condition is also a major factor in patient disability, with a significant impact on quality of life, by causing many obstacles in their daily activities and social functioning (2–4). This leads to an enormous economic burden among patients diagnosed with cLBP. A recently published analysis of real-world data estimated that cLBP accounted for $1.8 billion in societal costs within just the first year following diagnosis (4, 5).

The management of cLBP greatly depends on disease severity. Treatment guidelines within the US recommend different options for different severity categories. Lifestyle changes, psychotherapies, personalized exercise programs, and over-the-counter medications are first-line solutions for patients experiencing low-to-moderate severity of cLBP. Patients with moderate-to-severe pain require more complex management, usually combining non-opioid and opioid prescription medications. Invasive surgical procedures are also considered in certain cases, depending on the patient's profile and disease characteristics (6, 7).

Opioid medications are usually prescribed alongside other treatments. Although they are considered a highly effective option, persistent or inappropriate use is frequently associated with the occurrence of serious and/or severe treatment-related adverse events, including opioid use disorder (OUD). OUD is a complex behavioral and neurobiological syndrome which, if untreated, may lead to serious outcomes such as overdose and death. OUD increases the chances of severe and serious opioid-related adverse events occurring during treatment due to opioid overdosing and misuse, since most of them are dose-dependent. This is a major limitation that should be considered when choosing the right treatment option for patients with cLBP. The Centers for Disease Control and Prevention (CDC) published detailed guidelines on appropriate opioid treatment use with the aim of decreasing the risk of development of OUD and increasing patient safety (8, 9). In addition, the most recent initiative from the US Food and Drug Administration required opioid pain medicine manufacturers to update prescribing information regarding long-term use. The boxed warning emphasized that the risks of addiction, abuse/misuse, overdose, and death associated with opioids can occur at any dosage or duration and persist over the course of therapy (10).

The US Veterans Affairs/Department of Defense treatment guideline recommends buprenorphine as a safer alternative to standard opioids for chronic pain management because of a lower risk of overdose and misuse (11). Buprenorphine is an atypical opioid with partial agonism at µ-opioid receptors, a property that is thought to contribute to an improved safety profile relative to full agonists, particularly with respect to serious adverse events. The unique pharmacological characteristics of buprenorphine result in a ceiling effect on respiratory depression and translate to a lower risk of opioid-related euphoria, dependence, and overdose (12). The Drug Enforcement Administration (DEA) recognized buprenorphine as a substance with a moderate to low potential for physical and psychological dependence and categorized buprenorphine under schedule III (CIII) (9, 12–14). Therefore, buprenorphine also requires a personalized treatment plan adjusted to the patient's needs and preferences (14, 15). Another limitation is the low oral bioavailability of buprenorphine due to the first-pass hepatic metabolism. This is addressed through the development of transdermal patch and buccal film formulations for chronic pain management (12).

The main objective of this real-world evidence (RWE) study was to explore and compare the safety profiles of Belbuca® (buprenorphine buccal film) to buprenorphine transdermal patch and oral schedule II (CII) opioids in a population of commercially insured patients with cLBP who did not have a positive history of OUD. The treatment characteristics (utilization rates of rescue medications) were evaluated as secondary outcomes.

## Material and methods

### Data source

This retrospective RWE claims analysis was conducted using US insurance claims data from the Merative MarketScan® Commercial Claims Database. This database consists of medical and drug data for over 293 million individuals, encompassing employees and their spouses and dependents covered by employer-sponsored private health insurance in the US (16). The study was performed on insurance data claims captured in the period from January 1, 2019, to December 31, 2023. The study was conducted according to the Strengthening the Reporting of Observational studies in Epidemiology (STROBE) reporting recommendations for observational studies (17).

### Study population

The study population was identified based on specific inclusion/exclusion criteria to ensure a homogeneous sample of cLBP patients without a documented history of OUD who were prescribed Belbuca®, buprenorphine patch, or oral CII opioid [short-acting [SAO] or long-acting [LAO]] medications.

**Inclusion criteria:**

- Patients prescribed Belbuca®, buprenorphine patch, or oral CII opioids.
- Adult patients (age ≥ 18 years).
- At least two claims with a diagnosis of low back pain (LBP) based on the International Classification of Diseases—Clinical Modification (ICD-10-CM) codes (Supplementary Table S1) in the 6-month preindex period.
- Continuous healthcare coverage during the observational period.

**Exclusion criteria:**

- Patients prescribed Belbuca® or buprenorphine patch treatment within the CII opioid cohort during the observation period.
- Patients with any opioid prescription in the 6-month preindex period.
- OUD diagnosis based on the ICD-10-CM codes (Supplementary Table S2) during the 6-month preindex period.

To avoid an overlap between study cohorts, patients prescribed with Belbuca® and buprenorphine patch medications were allowed to have concomitant oral CII opioids during the follow-up period, while patients on CII opioids who received Belbuca® or buprenorphine patch prescriptions were excluded from the analysis. Therefore, the adverse event rates in buprenorphine-treated patients could be influenced by the concomitant use of CII opioid medications and potentially be overestimated.

### Study design

**Figure 1:**
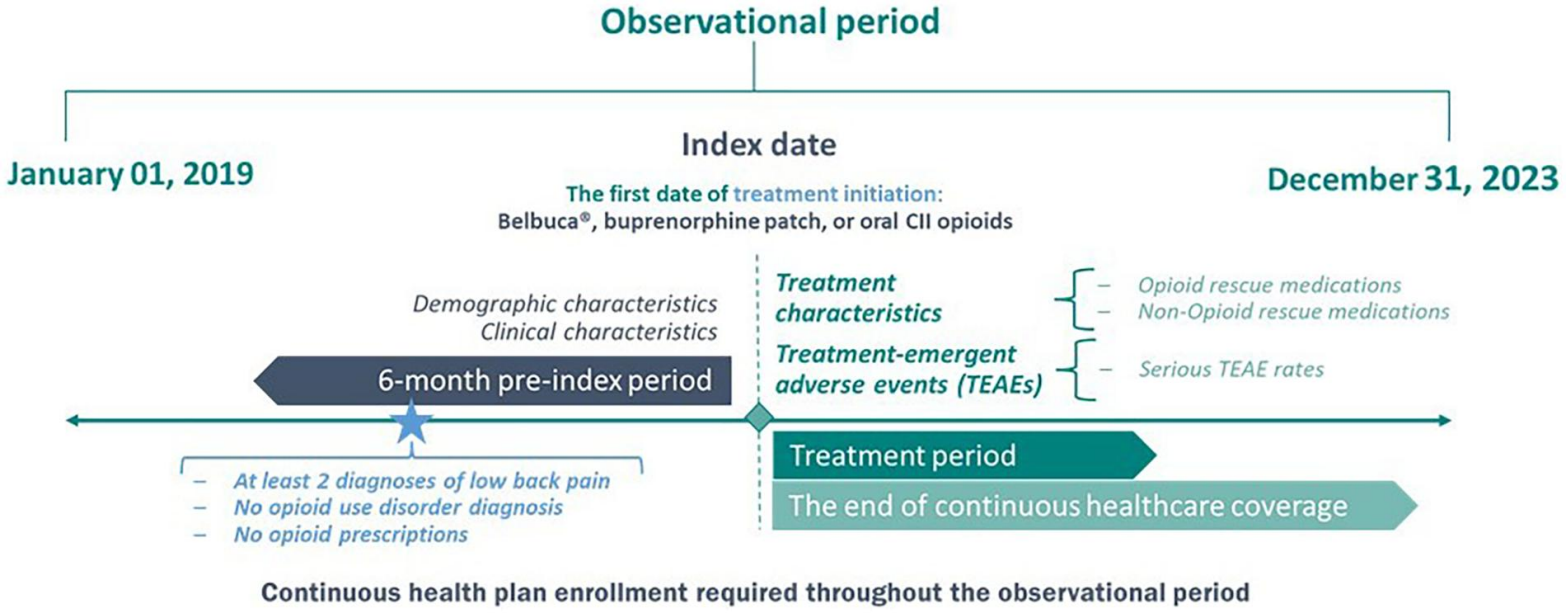
Study design.

Patients diagnosed with cLBP and treated with Belbuca®, buprenorphine patch, or oral CII opioids (SAO and LAO) were captured in the database based on the relevant National Drug Codes (NDCs) (Supplementary Tables S3, S4). The first date of Belbuca®, buprenorphine patch, or oral CII opioid prescription was assigned as the index date. The observational period consisted of a 6-month preindex period and a postindex period, defined by the final day of continuous healthcare and pharmaceutical coverage. Patients who had a diagnosis of OUD or any opioid prescription during the 6-month preindex period were excluded from the analysis. Demographic characteristics of the study sample were assessed on the index date, while clinical characteristics were evaluated during the 6-month preindex period. Based on the prescribed product on the index date, patients were stratified into cohorts—Belbuca®, buprenorphine patch, and oral CII opioid patients (SAO and LAO). The study design is presented in Figure 1.

### Outcome measures

The main outcome of the study was the incidence rate of serious treatment-emergent adverse events (TEAEs). Based on the most relevant buprenorphine or opioid evidence from published literature sources, the most common adverse events (≥5% rate), serious adverse events, adverse events leading to treatment discontinuation, and opioid-related adverse events were selected as TEAEs of interest for this analysis (18–27). In total, 44 relevant TEAEs were identified in the literature, stratified into the following categories:

- **Cardiovascular**: QT prolongation, hypotension, atrial fibrillation, coronary artery disease, hypertension.
- **Central nervous system (CNS)**: dizziness, somnolence, confusion, seizures, syncope, cerebrovascular accident, nervousness, visual discomfort, suicidal ideation, sleep disturbances.
- **OUD**: opioid abuse/dependence, opioid poisoning.
- **Hormonal**: adrenal insufficiency.
- **Musculoskeletal**: bone fractures, osteoarthritis.
- **Respiratory**: respiratory depression, pneumonia.
- **General**: headache, fatigue, allergic reactions, dehydration, dry mouth, xerostomia, sweating, hot flushes, sinusitis.
- **Gastrointestinal (GIT)**: nausea/vomiting, constipation, hepatotoxicity, cholecystitis, abdominal pain, diarrhea, anorexia/loss of appetite.
- **Skin toxicities**: cellulitis, pruritus, erythema, rash, skin irritation.
- **Urinary**: urinary discomfort.

**Figure 2:**
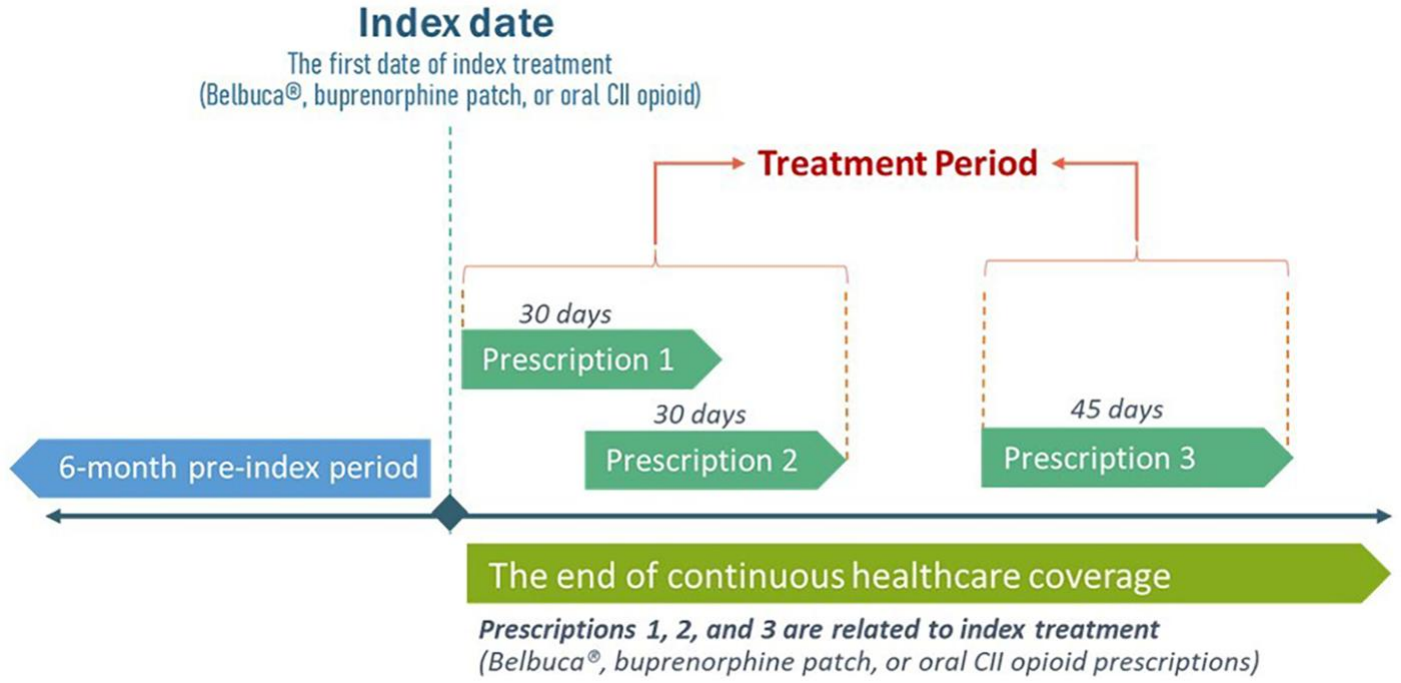
Treatment period definition scheme.

Potential TEAEs were captured in the database based on the ICD-10-CM codes (Supplementary Table S5). Only adverse events that occurred in the treatment period with index medication (Belbuca®, buprenorphine patch, or oral CII opioids) among patients without a positive history of the investigated adverse event during the preindex period were considered TEAEs. If the patient had a positive history of the investigated adverse event during the preindex period, it was considered that the corresponding TEAE was not associated with the index medication (as it had already occurred before the start of the cLBP treatment). Hence, the incidence rate of that adverse event was set to zero. The treatment period was defined as the sum of all periods during which patients were supplied with index medication, calculated using a starting date of prescription and days of supply for each prescription claim, excluding the treatment gaps but including the days of prescriptions that overlap. Although patients prescribed with Belbuca® and buprenorphine patch medications were allowed to have concomitant oral CII opioid use (SAO and LAO), only respective buprenorphine prescription claims were included when defining the treatment period. The scheme of treatment period definition is presented in Figure 2.

TEAEs were all relevant events captured within treatment periods, while serious TEAEs were defined as events claimed in inpatient or ED settings. As the postindex period duration differed between patients, TEAE rates were reported per 1,000 person-years and compared between study cohorts using the absolute incidence rate difference (IRD) and the incidence rate ratio (IRR) as outcome measures. The IRD represents the crude difference of the incidence rates observed among study cohorts (i.e., the incidence rate in cohort 1 minus the incidence rate in cohort 2), while the IRR is defined as a relative difference measure used to compare the incidence rates of adverse events (i.e., by dividing the incidence rate in cohort 1 with the incidence rate in cohort 2).

The treatment characteristics were observed as secondary study outcomes. The utilization rates of other rescue medications (opioid and non-opioid medications) were evaluated during the postindex period. Non-opioid rescue medications (NORMs) were classified as non-steroid anti-inflammatory drugs (NSAIDs), topiramate, duloxetine, and gabapentinoids based on treatment guidelines (6, 7). In addition, the numbers of opioid and non-opioid rescue medication prescriptions were estimated and reported.

As Belbuca® is the reference treatment for this study, analysis #1 compared Belbuca® with CII oral opioid cohorts, while analysis #2 compared patients prescribed with Belbuca® medication with those prescribed with buprenorphine patch medication.

### Statistical analysis

Continuous variables were summarized as means and standard deviations, while categorical variables were summarized as numbers and proportions of the sample. An independent t-test (continuous variables) and a chi-square test of independence (categorical variables) were performed to test the difference between the comparable cohorts. *P*-values lower than 0.05 implied statistical significance between the cohorts.

All serious TEAE rates and IRD values were reported per 1,000 person-years, while the IRR was reported as a rate ratio with 95% confidence intervals (95% CI). The incidence rate ratio test computed the IRD and IRR and explored the statistical significance of TEAE rate differences between the study cohorts. The *P*-value refers to the IRD result in case TEAE occurred in only one cohort, while significance related to the IRR result was used if the event was captured in both compared cohorts. Negative IRD values and an IRR less than 1 imply that the TEAE rate was lower in the referent cohort (Belbuca® in both analyses).

A propensity-score matching (PSM) analysis with the nearest-neighbor matching algorithm was performed to minimize selection bias and balance differences between the study cohorts. The demographic characteristics of patients observed on the index date and clinical characteristics observed throughout the preindex period were used as a basis for the matching process. The Belbuca® cohort was matched to the oral CII opioid cohort at a 1:4 ratio, with a caliper of 0.001 and random sampling (analysis #1). The following variables were used in the PSM analysis: age, gender, health plan, region of residence, Charlson comorbidity index (CCI) score, depression, insomnia, musculoskeletal pain, other neuropathies, fibromyalgia, mild liver disease, malignancy, diabetic neuropathy, peptic ulcer, spinal pain, moderate or severe liver disease, anxiety, and bipolar disorder. In addition, within analysis #2, patients consuming Belbuca® medication were matched to those consuming buprenorphine patch medication at a 1:1 ratio, with a caliper of 0.001, and random sampling. Patients' characteristics related to the health plan, CCI score, region of residence, and anxiety were used in the PSM process.

All statistical analyses were performed using the IBM Statistical Package for the Social Sciences (SPSS®), Statistical Analysis System (SAS®), and MedCalc® statistical software.

## Results

**Figure 3:**
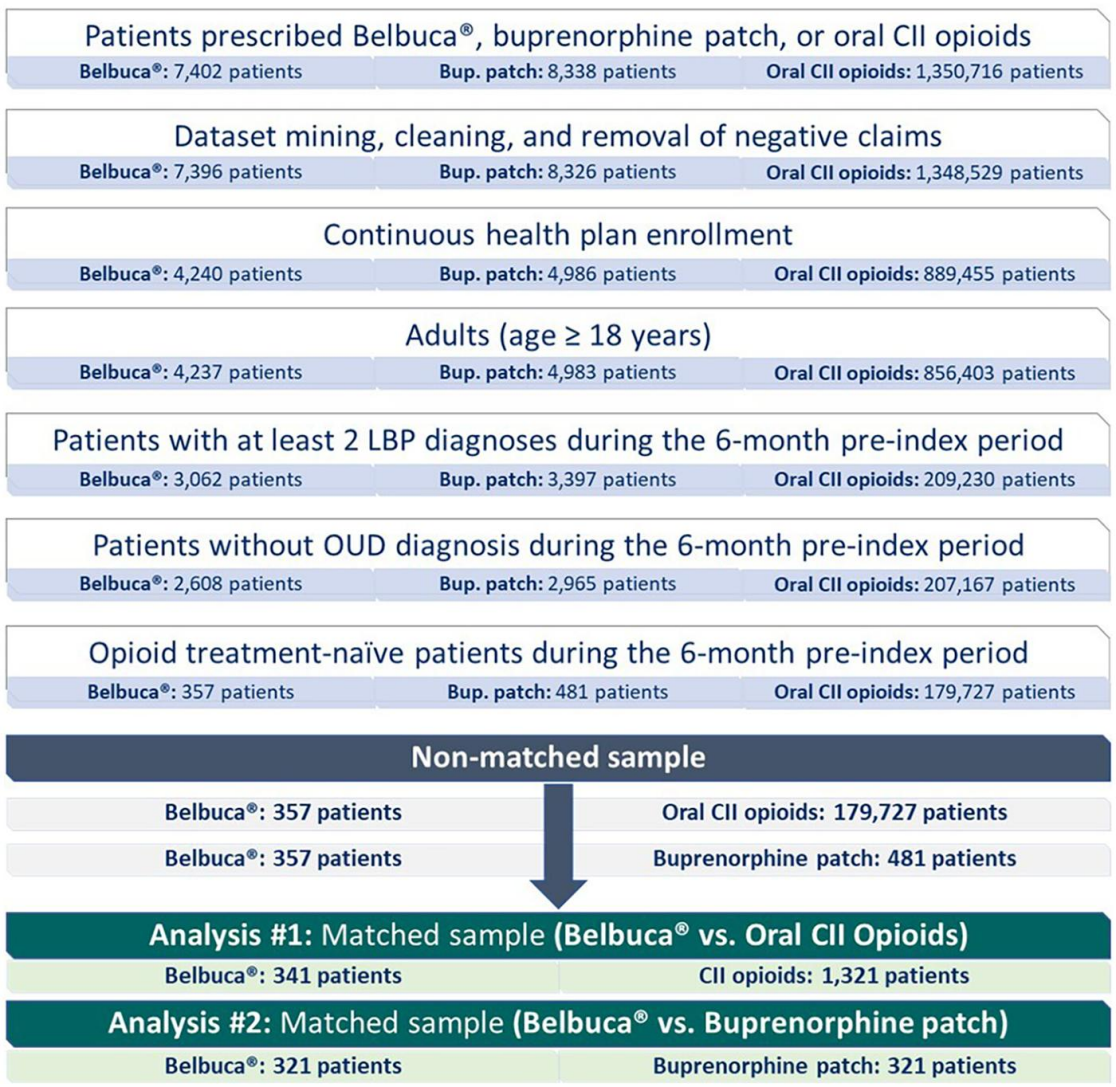
Patient selection flow diagram.

Out of 1,366,459 patients who were prescribed Belbuca®, buprenorphine patch, or oral CII opioids, 1,80,565 patients were identified in a final non-matched sample after inclusion/exclusion criteria were applied (357 in the Belbuca® cohort, 481 in the buprenorphine patch cohort, and 179,727 in the CII opioid cohort). After PSM, the final samples consisted of 1,662 patients in analysis #1 (341 patients prescribed Belbuca® matched to 1,321 patients prescribed CII opioids) and 642 patients in analysis #2 (321 patients prescribed Belbuca® matched to 321 patients prescribed buprenorphine patch). A patient selection flow diagram is presented in Figure 3.

### Analysis #1: Belbuca® vs. oral CII opioids

#### Non-matched population

A total non-matched sample consisted of 180,084 patients, with 357 patients initially prescribed Belbuca® and 179,727 patients initially prescribed oral CII opioids.

##### Demographic and clinical characteristics

Patients in the Belbuca® cohort were older (mean age 49.3 vs. 46.6 years, *p* < 0.001), with a higher proportion of females than in the CII opioid cohort (66.9% vs. 59.9%, *p* = 0.007). A higher rate of patients in the Belbuca® cohort was covered by a consumer-driven health plan (16.8% vs. 11.4%, *p* = 0.001). It was demonstrated that more patients in the Belbuca® cohort resided in the South region (70.0% vs. 45.4%, *p* < 0.001), while patients in the CII opioid cohort were predominantly living in the North-East (7.0% vs. 11.5%, *p* = 0.008), North-Central (13.2% vs. 24.7%, *p* < 0.001), and West (9.5% vs. 18.3%, *p* < 0.001) regions. All demographic characteristics of the non-matched analysis #1 sample are listed in Table 1.

**Table 1:** Analysis #1 (Belbuca® vs. oral CII opioids): demographic characteristics of non-matched patients.

Demographic Characteristics	Belbuca®	Oral CII opioids	*P*-value^a^
	(*N* = 357)	(*N* = 179,727)	
Age, mean (SD)	49.3 (9.9)	46.6 (12.3)	<0.001
Gender, *n* (%)			
Male	118 (33.1)	72,082 (40.1)	0.007
Female	239 (66.9)	107,645 (59.9)	0.007
Health plan, *n* (%)			
Basic/major medical	0 (0.0)	22 (0.0)	1.000
Comprehensive	14 (3.9)	4,352 (2.4)	0.066
Exclusive provider organization	5 (1.4)	1,378 (0.8)	0.206
Health maintenance organization	40 (11.2)	21,035 (11.7)	0.769
Non-capitated point-of-service	27 (7.6)	15,093 (8.4)	0.570
Point-of-service (POS) with capitation	1 (0.3)	555 (0.3)	1.000
Preferred provider organization	172 (48.2)	92,258 (51.3)	0.234
Consumer-driven health plan	60 (16.8)	20,500 (11.4)	0.001
High-deductible health plan	35 (9.8)	22,187 (12.3)	0.145
Unknown	3 (0.8)	2,347 (1.3)	0.638
Region, *n* (%)			
North-East	25 (7.0)	20,706 (11.5)	0.008
North-Central	47 (13.2)	44,379 (24.7)	<0.001
South	250 (70.0)	81,546 (45.4)	<0.001
West	34 (9.5)	32,851 (18.3)	<0.001
Unknown	1 (0.3)	245 (0.1)	0.386

a A chi-square test was performed for categorical variables and an independent t-test was performed for continuous variables.

Patients treated with Belbuca® had significantly higher CCI scores than those treated with oral CII opioids (1.1 vs. 0.7, *p* < 0.001), with a higher rate of patients in CCI 1, 3, and 4+ categories and a lower rate in the CCI category of 0 (all *p* < 0.050). With regard to the components of the CCI score, higher rates of congestive heart failure, peripheral vascular disease, cerebrovascular disease, chronic pulmonary disease, rheumatic disease, peptic ulcer disease, moderate or severe liver disease, and diabetes with and without chronic complications were noted in the Belbuca® cohort (all *p* < 0.050), while the rate of malignancies was higher in the oral CII opioid cohort (2.5% vs. 5.5%, *p* = 0.014). Significant differences between analysis #1 cohorts were observed in all mental health comorbidities and almost all other chronic pain comorbidities (except spine disorders). The rate of COVID infections was similar between the study cohorts. A list of all clinical characteristics in a non-matched analysis #1 sample is provided in Table 2.

**Table 2:** Analysis #1 (Belbuca® vs. oral CII opioids): clinical characteristics of non-matched patients.

Clinical Characteristics	Belbuca®	Oral CII opioids	*P*-value*^a^
	(*N* = 357)	(*N* = 179,727)	
Charlson comorbidity index			
0	193 (54.1)	125,327 (69.7)	<0.001
1	75 (21.0)	26,593 (14.8)	0.001
2	23 (6.4)	12,806 (7.1)	0.616
3	35 (9.8)	7,797 (4.3)	<0.001
4+	31 (8.7)	7,204 (4.0)	<0.001
Charlson comorbidity index, mean (SD)	1.1 (1.6)	0.7 (1.4)	<0.001
Charlson comorbidity index components			
Myocardial infarction	2 (0.6)	673 (0.4)	0.387
Congestive heart failure	9 (2.5)	1,902 (1.1)	0.007
Peripheral vascular disease	9 (2.5)	2,277 (1.3)	0.034
Cerebrovascular disease	13 (3.6)	3,160 (1.8)	0.007
Dementia	0 (0.0)	125 (0.1)	1.000
Chronic pulmonary disease	54 (15.1)	16,061 (8.9)	<0.001
Rheumatic disease	21 (5.9)	4,410 (2.5)	<0.001
Peptic ulcer disease	6 (1.7)	806 (0.4)	0.001
Mild liver disease	17 (4.8)	7,909 (4.4)	0.740
Moderate or severe liver disease	4 (1.1)	242 (0.1)	0.002
Diabetes without chronic complications	59 (16.5)	17,173 (9.6)	<0.001
Diabetes with chronic complications	55 (15.4)	11,122 (6.2)	<0.001
Hemiplegia or paraplegia	3 (0.8)	712 (0.4)	0.170
Renal disease	11 (3.1)	3,437 (1.9)	0.107
Malignancy	9 (2.5)	9,868 (5.5)	0.014
Metastatic solid tumor	2 (0.6)	1,569 (0.9)	0.775
AIDS/HIV	2 (0.6)	435 (0.2)	0.215
Mental disorders			
Anxiety	113 (31.7)	39,138 (21.8)	<0.001
Bipolar disorder	24 (6.7)	2,805 (1.6)	<0.001
Depression	106 (29.7)	24,351 (13.5)	<0.001
Sleep disorder	47 (13.2)	10,118 (5.6)	<0.001
Psychosis	12 (3.4)	2,995 (1.7)	0.013
Post-traumatic stress syndrome	13 (3.6)	3,385 (1.9)	0.015
Chronic pain–specific comorbidities			
Joint pain	171 (47.9)	70,835 (39.4)	0.001
Musculoskeletal disorders	289 (81.0)	117,365 (65.3)	<0.001
Diabetic neuropathy	19 (5.3)	3,164 (1.8)	<0.001
Other neuropathies	116 (32.5)	30,542 (17.0)	<0.001
Spine disorders	248 (69.5)	123,457 (68.7)	0.752
Fibromyalgia	69 (19.3)	16,734 (9.3)	<0.001
Other comorbidities			
COVID infection	20 (5.6)	7,919 (4.4)	0.271

a A chi-square test was performed for categorical variables and an independent t-test was performed for continuous variables.

#### Matched population

A total number of 1,662 patients were identified in the final sample of matched patients in analysis #1 (341 patients in the Belbuca® cohort and 1,321 patients in the CII opioid cohort). Patients were well balanced between the study cohorts, and there were no differences in observed patients' characteristics that could impact study outcome measures. A list of demographic characteristics is provided in Supplementary Table S6, while clinical characteristics are reported in Supplementary Table S7.

##### Treatment characteristics

The follow-up duration period was similar between the study cohorts (Table 3). Patients treated with Belbuca® had a significantly higher rate of non-opioid rescue medication utilization (60.7% vs. 45.1%, *p* < 0.001), driven by a higher proportion of patients who used topiramate (6.7% vs. 2.5%, *p* < 0.001), duloxetine (16.4% vs. 5.8%, *p* < 0.001), and gabapentinoids (39.6% vs. 20.0%, *p* < 0.001). There was a similar rate of NSAID utilization between the study cohorts (34.9% vs. 36.9%, *p* = 0.485). Across the total matched sample, several non-opioid rescue medication prescriptions were higher in the Belbuca® cohort than in the CII opioid cohort (5.0 vs. 2.9, *p* = 0.001). It was noted that 42.8% of patients treated with Belbuca® had at least one opioid prescription during the follow-up period (42.2% of patients had SAO and 1.8% had LAO), with an average of 2.5 prescriptions per patient.

**Table 3:** Analysis #1 (Belbuca® vs. oral CII opioids): treatment characteristics of matched patients.

Treatment Characteristics	Belbuca®	Oral CII opioids	*P*-value^a^
	(*N* = 341)	(*N* = 1,321)	
Follow-up duration (days), mean (SD)	207.1 (286.5)	195.2 (354.2)	0.518
Non-opioid rescue medications			
NSAIDs, mean (SD)			
Number of NSAID prescriptions^b^	1.6 (4.4)	1.3 (4.0)	0.293
Patients with at least one NSAID prescription, *n* (%)	119 (34.9)	488 (36.9)	0.485
Topiramate, mean (SD)			
Number of topiramate prescriptions^b^	0.3 (2.1)	0.2 (1.5)	0.170
Patients with at least one topiramate prescription, *n* (%)	23 (6.7)	33 (2.5)	<0.001
Duloxetine, mean (SD)			
Number of duloxetine prescriptions^b^	0.8 (3.4)	0.4 (2.8)	0.074
Patients with at least one duloxetine prescription, *n* (%)	56 (16.4)	76 (5.8)	<0.001
Gabapentinoids, mean (SD)			
Number of gabapentinoid prescriptions^b^	2.3 (6.1)	1.0 (3.8)	<0.001
Patients with at least one gabapentinoid prescription, *n* (%)	135 (39.6)	264 (20.0)	<0.001
NORMs, mean (SD)			
Number of NORM prescriptions^b^	5.0 (11.3)	2.9 (8.8)	0.001
Patients with at least one NORM prescription, *n* (%)	207 (60.7)	596 (45.1)	<0.001
Opioid rescue medications			
SAOs, mean (SD)			
Number of SAO prescriptions^b^	2.5 (6.3)	2.8 (5.2)	0.403
Patients with at least one SAO prescription, *n* (%)	144 (42.2)	1,319 (99.8)	<0.001
LAOs, mean (SD)			
Number of LAO prescriptions^b^	0.04 (0.40)	0.07 (0.79)	0.498
Patients with at least one LAO prescription, *n* (%)	6 (1.8)	15 (1.1)	0.358
Opioids (SAOs or LAOs), mean (SD)			
Number of opioid prescriptions^b^	2.5 (6.3)	2.8 (5.4)	0.366
Patients with at least one opioid prescription, *n* (%)	146 (42.8)	1,321 (100.0)	<0.001

a A chi-square test was performed for categorical variables and an independent t-test was performed for continuous variables.

b Across the total sample.

##### Serious treatment-emergent adverse events

No serious TEAEs occurred significantly more frequently in patients in the Belbuca® cohort than in those in the oral CII opioid cLBP cohort. In general, serious OUD (IRD -33.76 per 1,000 person-years, *p* = 0.032), gastrointestinal (IRR 0.35, *p* < 0.001), musculoskeletal (IRD −78.77 per 1,000 person-years, *p* < 0.001), and urinary (IRD −146.28 per 1,000 person-years, *p* < 0.001) TEAEs occurred more commonly in the CII opioid cohort. More specifically, significantly lower rates of serious seizures (IRR 0.11, *p* = 0.019), opioid abuse/dependence (IRD −33.76 per 1,000 person-years, *p* = 0.032), dehydration (IRR 0.13, *p* = 0.003), nausea and vomiting (IRR 0.30, *p* = 0.001), abdominal pain (IRR 0.25, *p* < 0.001), osteoarthritis (IRD −78.77 per 1,000 person-years, *p* = 0.001), and urinary discomfort (IRD −146.28 per 1,000 person-years, *p* < 0.001) were observed in the Belbuca® cohort. The total duration of the treatment period was numerically higher in the Belbuca® cohort than in the oral CII opioid cohort (135.99 vs. 88.87 person-years), indicating a longer time period for capturing TEAEs. All serious TEAE rates among the analysis #1 matched sample are listed in Table 4.

**Table 4:** Analysis #1 (Belbuca® vs. oral CII opioids): the incidence rates (per 1,000 person-years) and number of serious TEAEs during index treatments among matched cohorts.

Rate (number of events)	Belbuca® (*N* = 341)	CII opioids (*N* = 1,321)	Incidence rate difference (95% CI)	Incidence rate ratio (95% CI)	*P*-value^a^
Total duration of treatment period (person-years)	135.99	88.87	—	—	—
Cardiac adverse events	80.89 (11)	135.03 (12)	−54.14 (−139.64 to 31.36)	0.60 (0.24 to 1.48)	0.227
QT prolongation	0.00 (0)	0.00 (0)	—	—	—
Hypotension	0.00 (0)	22.50 (2)	−22.50 (47.72 to 2.71)	0.00 (0.00 to 3.48)	0.080
Atrial fibrillation	29.41 (4)	78.77 (7)	−49.36 (−108.48 to 9.78)	0.37 (0.08 to 1.47)	0.121
Coronary artery disease	36.77 (5)	11.25 (1)	25.52 (−18.16 to 69.19)	3.27 (0.37 to 154.55)	0.290
Hypertension	14.71 (2)	22.50 (2)	−7.79 (−43.46 to 27.86)	0.65 (0.05 to 9.02)	0.690
CNS adverse events	125.01 (17)	213.79 (19)	−88.79 (−195.76 to 18.19)	0.58 (0.29 to 1.19)	0.111
Dizziness	36.77 (5)	11.25 (1)	25.52 (−18.16 to 69.19)	3.27 (0.37 to 154.55)	0.290
Somnolence	0.00 (0)	11.25 (1)	−11.25 (−29.08 to 6.58)	0.00 (0.00 to 25.49)	0.216
Confusion	7.35 (1)	22.50 (2)	−15.15 (−46.03 to 15.73)	0.33 (0.01 to 6.28)	0.407
Seizures	7.35 (1)	67.51 (6)	−60.16 (−107.33 to −12.99)	0.11 (0.00 to 0.90)	0.019
Syncope	7.35 (1)	22.50 (2)	−15.15 (−46.03 to 15.73)	0.33 (0.01 to 6.28)	0.407
Cerebrovascular accident	22.06 (3)	45.01 (4)	−22.95 (−70.12 to 24.22)	0.49 (0.07 to 2.90)	0.372
Nervousness	0.00 (0)	0.00 (0)	—	—	—
Visual discomfort	0.00 (0)	0.00 (0)	—	—	—
Suicidal ideation	0.00 (0)	0.00 (0)	—	—	—
Sleep disturbances	44.12 (6)	33.76 (3)	10.36 (−43.12 to 63.85)	1.31 (0.28 to 8.08)	0.736
OUD adverse events	0.00 (0)	33.76 (3)	−33.76 (−64.64 to (−2.88))	0.00 (0.00–1.58)	0.032
OAD	0.00 (0)	33.76 (3)	−33.76 (−64.64 to −2.88)	0.00 (0.00 to 1.58)	0.032
Opioid poisoning	0.00 (0)	0.00 (0)	—	—	—
General adverse events	102.95 (14)	180.04 (16)	−77.09 (−174.04 to 20.56)	0.57 (0.26 to 1.25)	0.131
Headache	36.77 (5)	56.26 (5)	−19.49 (−75.87 to 36.88)	0.65 (0.15 to 2.84)	0.513
Fatigue	44.12 (6)	11.25 (1)	32.87 (−14.30 to 80.04)	3.92 (0.48 to 180.36)	0.195
Allergic reactions	7.35 (1)	0.00 (0)	7.35 (−10.48 to 25.18)	—	0.419
Dehydration	14.71 (2)	112.52 (10)	−97.81 (−159.58 to −36.06)	0.13 (0.01 to 0.61)	0.003
Dry mouth	0.00 (0)	0.00 (0)	—	—	—
Xerostomia	0.00 (0)	0.00 (0)	—	—	—
Sweating	0.00 (0)	0.00 (0)	—	—	—
Hot flushes	0.00 (0)	0.00 (0)	—	—	—
Sinusitis	0.00 (0)	0.00 (0)	—	—	—
GIT adverse events	382.39 (52)	1,080.22 (96)	−697.81 (−914.70 to −481.00)	0.35 (0.25 to 0.50)	<0.001
Nausea and vomiting	73.54 (10)	247.55 (22)	−174.01 (−274.90 to −73.20)	0.30 (0.13 to 0.65)	0.001
Constipation	95.60 (13)	101.27 (9)	−5.67 (−89.30 to 77.95)	0.94 (0.37 to 2.50)	0.886
Hepatotoxicity	0.00 (0)	0.00 (0)	—	—	—
Cholecystitis	36.77 (5)	67.51 (6)	−30.75 (−89.88 to 28.38)	0.54 (0.13 to 2.14)	0.330
Abdominal pain	139.72 (19)	562.62 (50)	−422.90 (−571.00 to −274.80)	0.25 (0.14 to 0.43)	<0.001
Diarrhea	36.77 (5)	78.77 (7)	−42.00 (−103.76 to 19.76)	0.47 (0.12 to 1.71)	0.203
Anorexia/loss of appetite	0.00 (0)	22.50 (2)	−22.50 (−47.72 to 2.71)	0.00 (0.00 to 3.48)	0.080
Hormonal adverse events	0.00 (0)	0.00 (0)	—	—	—
Adrenal insufficiency	0.00 (0)	0.00 (0)	—	—	—
Musculoskeletal adverse events	0.00 (0)	78.77 (7)	−78.77 (−125.94 to (−31.60)	0.00 (0.00 to 0.45)	0.001
Bone fractures	0.00 (0)	0.00 (0)	—	—	—
Osteoarthritis	0.00 (0)	78.77 (7)	−78.77 (−125.94 to −31.60)	0.00 (0.00 to 0.45)	0.001
Respiratory adverse events	66.18 (9)	67.51 (6)	−1.33 (−70.38 to 67.72)	0.98 (0.31 to 3.35)	0.957
Respiratory depression	22.06 (3)	0.00 (0)	22.06 (−8.82 to 52.94)	—	0.162
Pneumonia	44.12 (6)	67.51 (6)	−23.39 (−85.15 to 38.37)	0.65 (0.17 to 2.44)	0.472
Skin adverse events	80.89 (11)	146.28 (13)	−65.39 (−152.73 to 21.95)	0.55 (0.22 to 1.34)	0.154
Cellulitis	80.89 (11)	135.03 (12)	−54.14 (−139.64 to 31.36)	0.60 (0.24 to 1.48)	0.227
Pruritus	0.00 (0)	11.25 (1)	−11.25 (−29.08 to 6.58)	0.00 (0.00 to 25.49)	0.216
Erythema	0.00 (0)	0.00 (0)	—	—	—
Rash	0.00 (0)	0.00 (0)	—	—	—
Skin irritation	0.00 (0)	0.00 (0)	—	—	—
Urinary adverse events	0.00 (0)	146.28 (13)	−146.28 (−210.60 to −82.00)	0.00 (0.00 to 0.21)	<0.001
Urinary discomfort	0.00 (0)	146.28 (13)	−146.28 (−210.60 to −82.00)	0.00 (0.00 to 0.21)	<0.001

CNS, central nervous system; OUD, opioid use disorder; OAD, opioid abuse/dependence; GI, gastrointestinal; CI, confidence interval.

a An incidence rate ratio test was performed to assess statistical differences between the study cohorts. If the rate of adverse event was zero in one of the cohorts, the *p*-value reflected the difference between the study cohorts in the absolute incidence rate difference.

### Analysis #2: Belbuca® vs. buprenorphine patch

#### Non-matched population

A total non-matched sample in analysis #2 consisted of 838 patients, with 357 patients initially prescribed Belbuca® and 481 patients initially prescribed buprenorphine patch.

##### Demographic and clinical characteristics

There was a significant difference in mean age (49.3 years in Belbuca® vs. 51.0 years in buprenorphine patch, *p* = 0.009), while no differences were observed in gender distribution between the cohorts in analysis #2. Patients in the Belbuca® cohort were more commonly covered by consumer-driven health plans (16.8% vs. 8.5%, *p* < 0.001) and resided in the South region (70.0% vs. 54.9%, *p* < 0.001), while patients in the buprenorphine patch cohort predominantly resided in the North-Central area (13.2% vs. 22.2%, *p* = 0.001). All demographic characteristics of the analysis #2 non-matched sample are given in Table 5.

**Table 5:** Analysis #2 (Belbuca® vs. buprenorphine patch): demographic characteristics of non-matched patients.

Demographic Characteristics	Belbuca®	Bup. patch	*P*-value^a^
	(*N* = 357)	(*N* = 481)	
Age, mean (SD)	49.3 (9.9)	51.0 (9.7)	0.009
Gender, *n* (%)			
Male	118 (33.1)	153 (31.8)	0.703
Female	239 (66.9)	328 (68.2)	0.703
Health plan, *n* (%)			
Basic/major medical	0 (0.0)	0 (0.0)	—
Comprehensive	14 (3.9)	29 (6.0)	0.172
Exclusive provider organization	5 (1.4)	5 (1.0)	0.751
Health maintenance organization	40 (11.2)	69 (14.3)	0.181
Non-capitated point-of-service	27 (7.6)	38 (7.9)	0.857
POS with capitation	1 (0.3)	0 (0.0)	0.426
Preferred provider organization	172 (48.2)	249 (51.8)	0.304
Consumer-driven health plan	60 (16.8)	41 (8.5)	<0.001
High-deductible health plan	35 (9.8)	44 (9.1)	0.748
Unknown	3 (0.8)	6 (1.2)	0.740
Region, *n* (%)			
North-East	25 (7.0)	44 (9.1)	0.264
North-Central	47 (13.2)	107 (22.2)	0.001
South	250 (70.0)	264 (54.9)	<0.001
West	34 (9.5)	65 (13.5)	0.077
Unknown	1 (0.3)	1 (0.2)	1.000

a A chi-square test was performed for categorical variables and an independent t-test was performed for continuous variables.

Patients prescribed Belbuca® had a similar CCI score as those prescribed buprenorphine patch (1.1 vs. 1.2, *p* = 0.346), with a lower rate in the CCI category 2 compared with patients prescribed buprenorphine patch (6.4% vs. 12.3%, *p* = 0.005). In addition, mild liver disease (4.8% vs. 8.7%, *p* = 0.026) and malignancies (2.5% vs. 5.6%, *p* = 0.029) were components of the CCI, which was more common in patients prescribed buprenorphine patch. Significant differences were also noted in mental health disorders, with anxiety (31.7% vs. 40.5%, *p* = 0.008) and post-traumatic stress disorder (3.6% vs. 7.5%, *p* = 0.019) occurring more frequently in patients prescribed buprenorphine patch. From other pain comorbidities, a significant difference was noted only in the rate of fibromyalgia (19.3% vs. 25.8%, *p* = 0.028). A list of all clinical characteristics among analysis #2 non-matched samples is provided in Table 6.

**Table 6:** Analysis #2 (Belbuca® vs. buprenorphine patch): clinical characteristics of non-matched patients.

Clinical Characteristics	Belbuca®	Bup. patch	*P*-value^a^
	(*N* = 357)	(*N* = 481)	
Charlson comorbidity index			
0	193 (54.1)	247 (51.4)	0.437
1	75 (21.0)	96 (20.0)	0.709
2	23 (6.4)	59 (12.3)	0.005
3	35 (9.8)	30 (6.2)	0.056
4+	31 (8.7)	49 (10.2)	0.464
Charlson comorbidity index, mean (SD)	1.1 (1.6)	1.2 (1.8)	0.346
Charlson comorbidity index components			
Myocardial infarction	2 (0.6)	3 (0.6)	1.000
Congestive heart failure	9 (2.5)	6 (1.2)	0.169
Peripheral vascular disease	9 (2.5)	10 (2.1)	0.671
Cerebrovascular disease	13 (3.6)	11 (2.3)	0.245
Dementia	0 (0.0)	2 (0.4)	0.510
Chronic pulmonary disease	54 (15.1)	75 (15.6)	0.853
Rheumatic disease	21 (5.9)	42 (8.7)	0.122
Peptic ulcer disease	6 (1.7)	5 (1.0)	0.542
Mild liver disease	17 (4.8)	42 (8.7)	0.026
Moderate or severe liver disease	4 (1.1)	3 (0.6)	0.467
Diabetes without chronic complications	59 (16.5)	82 (17.0)	0.842
Diabetes with chronic complications	55 (15.4)	72 (15.0)	0.861
Hemiplegia or paraplegia	3 (0.8)	1 (0.2)	0.318
Renal disease	11 (3.1)	17 (3.5)	0.718
Malignancy	9 (2.5)	27 (5.6)	0.029
Metastatic solid tumor	2 (0.6)	7 (1.5)	0.315
AIDS/HIV	2 (0.6)	1 (0.2)	0.578
Mental disorders			
Anxiety	113 (31.7)	195 (40.5)	0.008
Bipolar disorder	24 (6.7)	22 (4.6)	0.177
Depression	106 (29.7)	142 (29.5)	0.957
Sleep disorder	47 (13.2)	81 (16.8)	0.144
Psychosis	12 (3.4)	17 (3.5)	0.892
Post-traumatic stress syndrome	13 (3.6)	36 (7.5)	0.019
Chronic pain–specific comorbidities			
Joint pain	171 (47.9)	256 (53.2)	0.127
Musculoskeletal disorders	289 (81.0)	389 (80.9)	0.977
Diabetic neuropathy	19 (5.3)	26 (5.4)	0.958
Other neuropathies	116 (32.5)	181 (37.6)	0.124
Spine disorders	248 (69.5)	347 (72.1)	0.399
Fibromyalgia	69 (19.3)	124 (25.8)	0.028
Other comorbidities			
COVID infection	20 (5.6)	18 (3.7)	0.201

a A chi-square test was performed for categorical variables and an independent t-test was performed for continuous variables.

#### Matched population

The final sample of matched patients in analysis #2 identified 642 patients (321 patients in both cohorts). The patient population was well balanced between the study cohorts, and there were no differences in patient characteristics that could impact study outcome measures. The demographic and clinical characteristics of the analysis #2 matched sample are given in Supplementary Tables S8, S9.

##### Treatment characteristics

The follow-up duration period was similar between the study cohorts (207.5 days in Belbuca® vs. 167.8 days in buprenorphine patch, *p* = 0.059). The rates of utilization of non-opioid rescue medication were similar between patients in the Belbuca® cohort and the buprenorphine patch cohort (61.4% vs. 59.2%, *p* = 0.572), with a higher number of non-opioid rescue medication prescriptions found among patients in the Belbuca® cohort (5.2 vs. 3.3, *p* = 0.010). The total number of non-opioid rescue medication prescriptions was driven by the utilization of gabapentinoids (2.4 prescriptions with Belbuca® vs. 1.4 prescriptions with buprenorphine patch, *p* = 0.012). There were no differences in the utilization of opioids observed between the study cohorts. Treatment characteristics are presented in Table 7.

**Table 7:** Analysis #2 (Belbuca® vs. buprenorphine patch): treatment characteristics of matched patients.

Treatment Characteristics	Belbuca®	Bup. patch	*P*-value^a^
	(*N* = 321)	(*N* = 321)	
Follow-up duration (days), mean (SD)	207.5 (290.9)	167.8 (236.9)	0.059
Non-opioid rescue medications			
NSAIDs, mean (SD)			
Number of NSAID prescriptions^b^	1.6 (4.4)	1.2 (3.2)	0.221
Patients with at least one NSAID prescription, *n* (%)	112 (34.9)	108 (33.6)	0.739
Topiramate, mean (SD)			
Number of topiramate prescriptions^b^	0.3 (2.1)	0.2 (1.4)	0.389
Patients with at least one topiramate prescription, *n* (%)	20 (6.2)	15 (4.7)	0.385
Duloxetine, mean (SD)			
Number of duloxetine prescriptions^b^	0.8 (3.5)	0.5 (1.6)	0.080
Patients with at least one duloxetine prescription, *n* (%)	55 (17.1)	48 (15.0)	0.452
Gabapentinoids, mean (SD)			
Number of gabapentinoid prescriptions^b^	2.4 (6.3)	1.4 (3.5)	0.012
Patients with at least one gabapentinoid prescription, *n* (%)	125 (38.9)	126 (39.3)	0.936
NORMs, mean (SD)			
Number of NORM prescriptions^b^	5.2 (11.6)	3.3 (6.2)	0.010
Patients with at least one NORM prescription, *n* (%)	197 (61.4)	190 (59.2)	0.572
Opioid rescue medications			
SAOs, mean (SD)			
Number of SAO prescriptions^b^	2.5 (6.3)	1.7 (4.5)	0.068
Patients with at least one SAO prescription, *n* (%)	134 (41.7)	124 (38.6)	0.421
LAOs, mean (SD)			
Number of LAO prescriptions^b^	0.04 (0.41)	0.02 (0.16)	0.445
Patients with at least one LAO prescription, *n* (%)	6 (1.9)	5 (1.6)	1.000
Opioids (SAOs or LAOs), mean (SD)			
Number of opioid prescriptions^b^	2.5 (6.4)	1.7 (4.5)	0.063
Patients with at least one opioid prescription, *n* (%)	136 (42.4)	128 (39.9)	0.521

a A chi-square test was performed for categorical variables and an independent t-test was performed for continuous variables.

b Across the total sample.

##### Serious treatment-emergent adverse events

The rates of serious cardiac (IRR 0.44, *p* = 0.025), OUD (IRD −193.54, *p* < 0.001), and skin-related (IRR 0.41, *p* = 0.011) TEAEs were significantly lower in the Belbuca® cohort, while the rate of gastrointestinal TEAEs (IRR 1.91, *p* = 0.015) was lower in the buprenorphine patch cohort. Serious coronary artery disease, headache, and cholecystitis occurred only in the Belbuca® cohort [all three IRDs 39.01 per 1,000 person-years (*p* = 0.035)]. However, the rates of serious QT prolongation (IRD −52.78 per 1,000 person-years, *p* = 0.009), hypertension (IRR 0.22, *p* = 0.043), confusion (IRR 0.10, *p* = 0.007), opioid abuse/dependence (IRD −184.75 per 1,000 person-years, *p* < 0.001), and cellulitis (IRR 0.41, *p* = 0.011) were significantly higher in the buprenorphine patch cohort. All serious TEAE rates in the analysis #2 matched sample are reported in Table 8.

**Table 8:** Analysis #2 (Belbuca® vs. buprenorphine patch): the incidence rates (per 1,000 person-years) and number of serious TEAEs during index treatments among matched cohorts.

Rate (number of events)	Belbuca® (*N* = 321)	Bup. patch (*N* = 321)	Incidence rate difference (95% CI)	Incidence rate ratio (95% CI)	*P*-value^a^
Total duration of treatment period (person-years)	128.19	113.67	—	—	—
Cardiac adverse events	85.81 (11)	193.54 (22)	−107.73 (−201.00 to −14.50)	0.44 (0.19 to 0.95)	0.025
QT prolongation	0.00 (0)	52.78 (6)	−52.78 (−92.56 to −13.01)	0.00 (0.00 to 0.75)	0.009
Hypotension	0.00 (0)	0.00 (0)	—	—	—
Atrial fibrillation	31.20 (4)	70.38 (8)	−39.18 (−95.42 to 17.07)	0.44 (0.10 to 1.66)	0.189
Coronary artery disease	39.01 (5)	0.00 (0)	39.01 (2.70 to 75.31)	—	0.035
Hypertension	15.60 (2)	70.38 (8)	−54.78 (−106.12 to −3.43)	0.22 (0.02 to 1.11)	0.043
CNS adverse events	124.82 (16)	202.34 (23)	−77.52 (−178.92 to 23.87)	0.62 (0.30 to 1.22)	0.139
Dizziness	31.20 (4)	52.78 (6)	−21.58 (−72.93 to 29.76)	0.59 (0.12 to 2.49)	0.433
Somnolence	0.00 (0)	0.00 (0)	—	—	—
Confusion	7.80 (1)	79.18 (9)	−71.38 (−122.72 to −20.03)	0.10 (0.00 to 0.71)	0.007
Seizures	7.80 (1)	0.00 (0)	7.80 (−8.44 to 24.04)	—	0.346
Syncope	7.80 (1)	8.80 (1)	−1.00 (−23.96 to 21.97)	0.89 (0.01 to 69.61)	0.940
Cerebrovascular accident	23.40 (3)	17.59 (2)	5.81 (−30.50 to 42.11)	1.33 (0.15 to 15.93)	0.783
Nervousness	0.00 (0)	0.00 (0)	—	—	—
Visual discomfort	0.00 (0)	8.80 (1)	−8.80 (−25.03 to 7.44)	0.00 (0.00 to 34.58)	0.288
Suicidal ideation	0.00 (0)	17.59 (2)	−17.59 (−40.56 to 5.37)	0.00 (0.00 to 4.72)	0.133
Sleep disturbances	46.81 (6)	17.59 (2)	29.22 (−16.71 to 75.14)	2.66 (0.48 to 26.95)	0.238
OUD adverse events	0.00 (0)	193.54 (22)	−193.54 (−269.70 to −117.40)	0.00 (0.00 to 0.16)	<0.001
OAD	0.00 (0)	184.75 (21)	−184.75 (−259.20 to −110.30)	0.00 (0.00 to 0.17)	<0.001
Opioid poisoning	0.00 (0)	8.80 (1)	−8.80 (−25.03 to 7.44)	0.00 (0.00 to 34.58)	0.288
General adverse events	109.22 (14)	70.38 (8)	38.83 (−37.32 to 114.99)	1.55 (0.61 to 4.27)	0.329
Headache	39.01 (5)	0.00 (0)	39.01 (2.70 to 75.31)	—	0.035
Fatigue	39.01 (5)	8.80 (1)	30.21 (−9.56 to 69.98)	4.43 (0.50 to 209.70)	0.162
Allergic reactions	7.80 (1)	0.00 (0)	7.80 (−8.44 to 24.04)	—	0.346
Dehydration	23.40 (3)	26.39 (3)	−2.99 (−42.76 to 36.78)	0.89 (0.12 to 6.62)	0.888
Dry mouth	0.00 (0)	0.00 (0)	—	—	—
Xerostomia	0.00 (0)	0.00 (0)	—	—	—
Sweating	0.00 (0)	17.59 (2)	−17.59 (−40.56 to 5.37)	0.00 (0.00 to 4.72)	0.133
Hot flushes	0.00 (0)	0.00 (0)	—	—	—
Sinusitis	0.00 (0)	17.59 (2)	−17.59 (−40.56 to 5.37)	0.00 (0.00 to 4.72)	0.133
GIT adverse events	335.45 (43)	175.95 (20)	159.50 (30.60 to 288.40)	1.91 (1.10 to 3.42)	0.015
Nausea and vomiting	70.21 (9)	87.97 (10)	−17.76 (−88.54 to 53.01)	0.80 (0.29 to 2.19)	0.630
Constipation	62.41 (8)	17.59 (2)	44.82 (−6.53 to 96.16)	3.55 (0.71 to 34.29)	0.096
Hepatotoxicity	0.00 (0)	0.00 (0)	—	—	—
Cholecystitis	39.01 (5)	0.00 (0)	39.01 (2.70 to 75.31)	—	0.035
Abdominal pain	132.62 (17)	61.58 (7)	71.04 (−8.51 to 150.58)	2.15 (0.85 to 6.14)	0.083
Diarrhea	31.20 (4)	8.80 (1)	22.40 (−13.90 to 58.71)	3.55 (0.35 to 174.68)	0.269
Anorexia/loss of appetite	0.00 (0)	0.00 (0)	—	—	—
Hormonal adverse events	0.00 (0)	0.00 (0)	—	—	—
Adrenal insufficiency	0.00 (0)	0.00 (0)	—	—	—
Musculoskeletal adverse events	0.00 (0)	0.00 (0)	—	—	—
Bone fractures	0.00 (0)	0.00 (0)	—	—	—
Osteoarthritis	0.00 (0)	0.00 (0)	—	—	—
Respiratory adverse events	70.21 (9)	61.58 (7)	8.63 (−56.32 to 73.57)	1.14 (0.38 to 3.60)	0.805
Respiratory depression	23.40 (3)	43.99 (5)	−20.59 (−66.51 to 25.34)	0.53 (0.08 to 2.73)	0.408
Pneumonia	46.81 (6)	17.59 (2)	29.22 (−16.71 to 75.14)	2.66 (0.48 to 26.95)	0.238
Skin adverse events	85.81 (11)	211.14 (24)	−125.33 (−221.40 to −29.30)	0.41 (0.18 to 0.86)	0.011
Cellulitis	85.81 (11)	211.14 (24)	−125.33 (−221.40 to −29.30)	0.41 (0.18 to 0.86)	0.011
Pruritus	0.00 (0)	0.00 (0)	—	—	—
Erythema	0.00 (0)	0.00 (0)	—	—	—
Rash	0.00 (0)	0.00 (0)	—	—	—
Skin Irritation	0.00 (0)	0.00 (0)	—	—	—
Urinary adverse events	0.00 (0)	0.00 (0)	—	—	—
Urinary discomfort	0.00 (0)	0.00 (0)	—	—	—

CNS, central nervous system; OUD, opioid use disorder; OAD, opioid abuse/dependence; GIT, gastrointestinal; CI, confidence interval.

a An incidence rate ratio test assessed statistical differences between the study cohorts. If the rate of adverse event was zero in one of the cohorts, the *p*-value reflected the difference between the study cohorts in the absolute incidence rate difference.

## Discussion

This retrospective RWE claims analysis showed a favorable safety and tolerability profile of Belbuca® compared with oral CII opioids and buprenorphine transdermal patches in a matched population of commercially insured patients with cLBP without a positive history of OUD. Out of 44 relevant serious TEAEs observed during treatment, Belbuca® showed significantly lower rates of 7 serious TEAEs compared with oral CII opioids. Notably, no serious TEAEs occurred at a significantly higher rate in the Belbuca® cohort. Patients in the Belbuca® cohort had lower opioid utilization rates but a higher utilization of non-opioid rescue medications (driven by gabapentinoids). In the matched analysis #2, patients in the Belbuca® cohort had significantly lower rates of five serious TEAEs but significantly higher rates of three serious TEAEs. Serious QT prolongation and opioid abuse/dependence occurred only in the buprenorphine patch cohort. Conversely, serious coronary artery disease, headache, and cholecystitis were serious TEAEs observed exclusively in the Belbuca® cohort. Opioid utilization was similar between the study cohorts; however, patients in the Belbuca® group used more non-opioid rescue medications, primarily because of increased gabapentinoid use. Follow-up was ∼50 days longer in the Belbuca® cohort than in the transdermal patch cohort but lacked statistical significance (*p* = 0.059).

This study is an addendum to previously published retrospective insurance claims analyses exploring Belbuca® safety for cLBP management in a real-world setting. Stanicic et al. (28) reported a better safety profile of commercially insured patients with cLBP treated with Belbuca® than with buprenorphine patch or oral opioid medications. The Belbuca® cohort had significantly lower serious rates of 13 out of 44 observed TEAEs compared with oral CII opioids (all *p* ≤ 0.024) and only a higher rate of serious dizziness (IRR 3.17, *p* = 0.024). Compared with the buprenorphine patch cohort, Belbuca® was associated with a significantly lower rate of five serious TEAEs (all *p* ≤ 0.018), including opioid abuse/dependence (IRR 0.04, *p* < 0.001). However, it showed significantly higher rates of serious cholecystitis (IRD 52.17, *p* = 0.035) and suicidal ideation (IRD 156.50, *p* < 0.001), both driven by a single outlier case per event (28). A real-world analysis by Grbic et al. (29) explored the safety profiles of Belbuca®, buprenorphine patch, and oral CII opioids in Medicare patients diagnosed with cLBP. The safety profile comparison between Belbuca® and oral CII opioids revealed significantly lower rates of serious constipation, fatigue, osteoarthritis, and urinary discomfort among patients prescribed with Belbuca® (all *p* ≤ 0.043). Notably, no serious TEAEs occurred at significantly higher rates in the Belbuca® cohort compared with the oral CII opioid cohort. Compared with the buprenorphine patch cohort, the Belbuca® cohort showed significantly higher rates of serious osteoarthritis and confusion (IRDs 867.30 and 462.56 per 1,000 person-years, respectively; both *p* < 0.050) but lower rates of serious pneumonia, dehydration, opioid abuse/dependence, abdominal pain, and loss of appetite (all *p* < 0.050) (29). The findings from this analysis are in line with those from the previously published literature on this topic. Because OUD may lead to opioid overdose and most opioid-related adverse events are dose-dependent, observing TEAE occurrence among patients without a positive OUD history provides another objective overview and a reliable comparison of safety profiles of Belbuca®, buprenorphine transdermal patch, and oral CII opioids prescribed for cLBP management.

A literature review by Hale et al. (30) summarized the available evidence regarding buprenorphine safety in chronic pain management and provided a critical overview in contrast to conventional opioid medications. Published evidence confirmed that buprenorphine has much lower abuse potential and substantially less overdose-related deaths than CII opioids. In addition to OUD, buprenorphine reduces the risk of death due to respiratory depression associated with opioid use compared with other conventional medications in this category. Buprenorphine transdermal patches even showed an approximately 80 times lower rate of respiratory depression than transdermal fentanyl. Constipation was reported in up to 23% of patients who received CII LAOs, compared with only 4% of patients on Belbuca® and 13% on the buprenorphine transdermal patch. The rates of other gastrointestinal and general adverse events (nausea, vomiting, constipation, headache, dizziness, somnolence, anxiety, and dry mouth) were also numerically lower in patients on buprenorphine (including both Belbuca® and transdermal patches) compared with those on oxycodone, hydromorphone, and oxymorphone (30).

The safety benefits of Belbuca® compared with CII opioids have also been demonstrated in clinical trial settings. The administration of Belbuca®, including the maximum available prescription dose, did not cause a significant decrease in respiratory drive compared with placebo, while the utilization of oxycodone (a specific CII opioid comparator) showed a significant dose-dependent reduction in respiratory drive. In addition, Belbuca® use was associated with delayed changes in pupil diameter compared with oxycodone, which is linked to a lower risk of drug liking—a key factor in the development of OUD (31, 32).

### Strengths and limitations

This retrospective RWE claims analysis is an update of previously conducted real-world evidence studies among commercially insured and Medicare patients with cLBP that evaluated the safety profiles of CIII buprenorphine (Belbuca® and buprenorphine patch) and oral CII opioid treatments (28, 29). This analysis was performed using the most recent retrospective claims database within the sample of patients with cLBP who did not have a positive history of OUD before the treatment initiation. The comprehensive analysis explored rates from the exhaustive list of serious TEAEs and compared them between Belbuca® vs. oral CII opioids and Belbuca® vs. buprenorphine transdermal patches. Patients were required to have continuous insurance coverage during the pre- and postindex periods, which ensured a trustworthy longitudinal observation and completeness of data. PSM was employed to provide a valid between-group comparison by ensuring a homogeneous pool of patients with cLBP regarding their demographic and clinical characteristics and to avoid the impact of such covariates on safety outcomes. In addition, the TEAE events captured during the treatment period were not included in the incidence rate calculation for patients with a positive history of investigated events in the preindex period. This approach was employed to enhance the connection between the index treatment and relevant TEAEs.

However, the limitations of the study should be considered when interpreting the results. First, the main limitation is related to the restrictions of coding systems due to the nature and characteristics of real-world insurance claims. These data are primarily collected for billing purposes. Henceforth, many data entry errors may have occurred (miscoding, duplicate claims, or negative-input claims). The impact of this limitation was reduced to a minimum by performing thorough data cleaning, following a careful patient selection process, and adopting bias-controlling methods such as PSM. Second, the chronicity and severity of LBP could not be precisely determined in the database, which prevented the authors from comparing patients with LBP with those with similar pain severity. Chronicity was assumed in patients who had LBP claimed at least 2 times during the preindex period. The third limitation is associated with the underlying causes of TEAEs. Although we made an attempt to enhance the association between the treatment and TEAEs by capturing only those events that occurred in patients who were prescribed with the index medications and in patients with no history of investigated events during the preindex period, we were unable to confirm in the insurance claim database that a particular event was directly caused by the treatment. In addition, as patients prescribed with buprenorphine were allowed to have concomitant CII opioid treatment, there is a possibility that some of the TEAEs were caused by the concomitant use of CII opioids in the buprenorphine cohort. The impact of opioids on the positive history and occurrence of TEAEs was minimized by the requirement of opioid washout during the 6-month preindex period. This study was conducted in a sample of opioid-naïve patients with cLBP, excluding opioid-tolerant patients who required long-term opioid therapy. Hence, the generalizability of the study findings may be limited in standard clinical practice. In addition, this study included the COVID-19 pandemic, which may have influenced treatment patterns and safety outcomes.

## Conclusion

This RWE claims analysis showed better safety outcomes in patients with cLBP treated with Belbuca compared with those treated with oral CII opioids. Lower rates of serious TEAEs were reported for seizures, opioid abuse/dependence, dehydration, nausea/vomiting, abdominal pain, osteoarthritis, and urinary discomfort. No serious TEAEs were significantly more frequent in patients prescribed Belbuca® than in those prescribed oral CII opioids. In addition, no OUD-related serious TEAEs were identified during Belbuca® treatment in this study sample. Belbuca® was more frequently used alongside non-opioid rescue medications compared with oral CII opioids, while lower opioid utilization rates were also demonstrated over a similar follow-up duration.

This analysis also showed that Belbuca® had significantly lower rates of serious QT prolongation, hypertension, confusion, opioid abuse/dependence, and cellulitis compared with buprenorphine patch. In addition, there were no serious OUD-related TEAEs in the Belbuca® cohort of patients. Only serious coronary artery disease, headache, and cholecystitis were observed more frequently in patients prescribed with Belbuca® than in those prescribed buprenorphine patch. Follow-up duration and the rates of non-opioid and opioid rescue medication utilization were similar between the Belbuca® and buprenorphine patch cohorts.

The study findings suggest that Belbuca® may have a favorable safety profile relative to oral CII opioids and buprenorphine patch treatments in patients with cLBP without a positive history of OUD. The head-to-head randomized clinical trial should be performed as the gold standard to provide clinical evidence supporting the findings from this study. In the absence of clinical trials data, future RWE studies should investigate the safety profile of Belbuca® in a larger sample of patients with cLBP within the US healthcare setting to provide more generalizable results.

## Data Availability

The datasets presented in this article are not readily available because the datasets generated during and/or analyzed during the current study are not publicly available due to a confidentiality agreement between ZRx Outcomes Research Inc. and the data provider. Requests to access the datasets should be directed to Merative MarketScan®, marketscan.support@merative.com.
