# Supplementary material for: Treatment characteristics and safety profiles of Belbuca®, buprenorphine patch, and oral schedule II opioids among chronic low back pain patients without a positive history of opioid-use disorder: a retrospective US commercial claims analysis
Source: Front Pain Res (Lausanne). 2026 Jun 30;7:1764842. doi: 10.3389/fpain.2026.1764842 (PMC13365331; doi:10.3389/fpain.2026.1764842)
Supplement: Supplementary file 1 [file Table1.docx]

Supplementary Material

**Table S1.** ICD-10-CM codes related to low back pain

| **ICD-10-CM** | **Description** |
| --- | --- |
| **M43.06** | Spondylolysis lumbar region |
| **M43.07** | Spondylolysis lumbosacral region |
| **M43.08** | Spondylolysis sacral and sacrococcygeal region |
| **M43.16** | Spondylolisthesis lumbar region |
| **M43.17** | Spondylolisthesis lumbosacral region |
| **M43.18** | Spondylolisthesis sacral and sacrococcygeal region |
| **M43.27** | Fusion of spine, lumbosacral region |
| **M43.28** | Fusion of spine, sacral and sacrococcygeal region |
| **M45.6** | Ankylosing spondylitis lumbar region |
| **M45.7** | Ankylosing spondylitis of lumbosacral region |
| **M45.8** | Ankylosing spondylitis sacral and sacrococcygeal region |
| **M46.06** | Spinal enthesopathy lumbar region |
| **M46.07** | Spinal enthesopathy lumbosacral region |
| **M46.08** | Spinal enthesopathy sacral and sacrococcygeal region |
| **M46.46** | Discitis, unspecified …… lumbar region |
| **M46.47** | Discitis, unspecified …… lumbosacral region |
| **M46.48** | Discitis, unspecified …… sacral and sacrococcygeal region |
| **M47.16** | Other spondylosis with myelopathy…… lumbar region |
| **M47.816** | Spondylosis without myelopathy or radiculopathy …… lumbar region |
| **M47.817** | Spondylosis without myelopathy or radiculopathy …… lumbosacral region |
| **M47.818** | Spondylosis without myelopathy or radiculopathy …… sacral and sacrococcygeal region |
| **M48.06** | Spinal stenosis, lumbar region |
| **M48.061** | Spinal stenosis, lumbar region…… without neurogenic claudication |
| **M48.062** | Spinal stenosis, lumbar region…… with neurogenic claudication |
| **M48.07** | Spinal stenosis …… lumbosacral region |
| **M48.08** | Spinal stenosis …… sacral and sacrococcygeal region |
| **M51.06** | Intervertebral disc disorders with myelopathy, lumbar region |
| **M51.26** | Other intervertebral disc displacement, lumbar region |
| **M51.27** | Other intervertebral disc displacement, lumbosacral region |
| **M51.36** | Other intervertebral disc degeneration, lumbar region |
| **M51.37** | Other intervertebral disc degeneration, lumbosacral region |
| **M51.46** | Schmorl's nodes…… lumbar region |
| **M51.47** | Schmorl's nodes…… lumbosacral region |
| **M51.86** | Other intervertebral disc disorders, lumbar region |
| **M51.87** | Other intervertebral disc disorders, lumbosacral region |
| **M53.2X8** | Spinal instabilities…… sacral and sacrococcygeal region |
| **M53.3** | Sacrococcygeal disorders, not elsewhere classified |
| **M54.16** | Radiculopathy…… lumbar region |
| **M54.17** | Radiculopathy…… lumbosacral region |
| **M54.30** | Sciatica…… unspecified side |
| **M54.31** | Sciatica…… right side |
| **M54.32** | Sciatica…… left side |
| **M54.40** | Lumbago with sciatica…… unspecified side |
| **M54.41** | Lumbago with sciatica…… right side |
| **M54.42** | Lumbago with sciatica…… left side |
| **M54.5** | Low back pain |
| **M54.50** | …… unspecified |
| **M54.51** | Vertebrogenic low back pain |
| **M54.59** | Other low back pain |
| **M96.1** | Postlaminectomy syndrome, not elsewhere classified |
| **M99.03** | Segmental and somatic dysfunction…… of lumbar region |
| **M99.04** | Segmental and somatic dysfunction…… of sacral region |
| **M99.13** | Subluxation complex (vertebral)…… of lumbar region |
| **M99.14** | Subluxation complex (vertebral)…… of sacral region |

**Table S2.** ICD-10-CM codes related to opioid use disorder

| **Opioid use disorder** | **ICD-10-CM codes** |  |
| --- | --- | --- |
| Opioid abuse and dependence | F11, F11.1, F11.10, F11.11, F11.12, F11.120, F11.121, F11.122, F11.129, F11.13, F11.14, F11.15, F11.150, F11.151, F11.159, F11.18, F11.181, F11.182, F11.188, F11.19, F11.2, F11.20, F11.21, F11.22, F11.220, F11.221, F11.222, F11.229, F11.23, F11.24, F11.25, F11.250, F11.251, F11.259, F11.28, F11.281, F11.282, F11.288, F11.29, F11.9, F11.90, F11.91, F11.92, F11.920, F11.921, F11.922, F11.929, F11.93, F11.94, F11.95, F11.950, F11.951, F11.959, F11.98, F11.981, F11.982, F11.988, F11.99 |  |
| Opioid poisoning | T40, T40.0, T40.0X, T40.0X1, T40.0X1A, T40.0X1D, T40.0X1S, T40.0X2, T40.0X2A, T40.0X2D, T40.0X2S, T40.0X3, T40.0X3A, T40.0X3D, T40.0X3S, T40.0X4, T40.0X4A, T40.0X4D, T40.0X4S, T40.0X5, T40.0X5A, T40.0X5D, T40.0X5S, T40.0X6, T40.0X6A, T40.0X6D, T40.0X6S, T40.2, T40.2X, T40.2X1, T40.2X1A, T40.2X1D, T40.2X1S, T40.2X2, T40.2X2A, T40.2X2D, T40.2X2S, T40.2X3, T40.2X3A, T40.2X3D, T40.2X3S, T40.2X4, T40.2X4A, T40.2X4D, T40.2X4S, T40.2X5, T40.2X5A, T40.2X5D, T40.2X5S, T40.2X6, T40.2X6A, T40.2X6D, T40.2X6S, T40.3, T40.3X, T40.3X1, T40.3X1A, T40.3X1D, T40.3X1S, T40.3X2, T40.3X2A, T40.3X2D, T40.3X2S, T40.3X3, T40.3X3A, T40.3X3D, T40.3X3S, T40.3X4, T40.3X4A, T40.3X4D, T40.3X4S, T40.3X5, T40.3X5A, T40.3X5D, T40.3X5S, T40.3X6, T40.3X6A, T40.3X6D, T40.3X6S, T40.4, T40.41, T40.411, T40.411A, T40.411D, T40.411S, T40.412, T40.412A, T40.412D, T40.412S, T40.413, T40.413A, T40.413D, T40.413S, T40.414, T40.414A, T40.414D, T40.414S, T40.415, T40.415A, T40.415D, T40.415S, T40.416, T40.416A, T40.416D, T40.416S, T40.42, T40.421, T40.421A, T40.421D, T40.421S, T40.422, T40.422A, T40.422D, T40.422S, T40.423, T40.423A, T40.423D, T40.423S, T40.424, T40.424A, T40.424D, T40.424S, T40.425, T40.425A, T40.425D, T40.425S, T40.426, T40.426A, T40.426D, T40.426S, T40.49, T40.491, T40.491A, T40.491D, T40.491S, T40.492, T40.492A, T40.492D, T40.492S, T40.493, T40.493A, T40.493D, T40.493S, T40.494, T40.494A, T40.494D, T40.494S, T40.495, T40.495A, T40.495D, T40.495S, T40.496, T40.496A, T40.496D, T40.496S, T40.6, T40.60, T40.601, T40.601A, T40.601D, T40.601S, T40.602, T40.602A, T40.602D, T40.602S, T40.603, T40.603A, T40.603D, T40.603S, T40.604, T40.604A, T40.604D, T40.604S, T40.605, T40.605A, T40.605D, T40.605S, T40.606, T40.606A, T40.606D, T40.606S, T40.69, T40.691, T40.691A, T40.691D, T40.691S, T40.692, T40.692A, T40.692D, T40.692S, T40.693, T40.693A, T40.693D, T40.693S, T40.694, T40.694A, T40.694D, T40.694S, T40.695, T40.695A, T40.695D, T40.695S, T40.696, T40.696A, T40.696D, T40.696S |  |
|  |  |  |

**Table S3.** The relevant NDC codes related to Belbuca® and buprenorphine transdermal patches

| **Description** | **NDC codes** |
| --- | --- |
| **Belbuca®** | 55700086760, 59385002160, 59385002260, 59385002360, 59385002401, 59385002460, 59385002501, 59385002560, 59385002601, 59385002660, 59385002760, 63481016160, 63481020760, 63481034860, 63481051960, 63481068560, 63481082060, 63481095260 |
| **Buprenorphine Patch** | 00093323921, 00093323940, 00093360021, 00093360040, 00093360121, 00093360140, 00093360221, 00093360240, 00093360321, 00093360340, 00093365621, 00093365640, 00093365721, 00093365740, 00093365821, 00093365840, 00093365921, 00093365940, 35356060504, 35356060604, 35356060704, 42858035340, 42858049340, 42858058640, 42858075040, 42858083940, 54569632500, 54569632600, 55700056804, 55700057904, 59011075004, 59011075104, 59011075204, 59011075704, 59011075804, 60505707505, 60505707605, 60505707705, 60505707805, 60505707905, 69238120202, 69238120302, 69238120402, 69238120502, 69238150502 |

**Table S4.** The list of relevant oral CII opioids (SAO and LAO)

| **Category** | **Medications** |
| --- | --- |
| **Oral Schedule II  Opioids (SAO, LAO)** | Codeine Phosphate  Codeine Sulfate  Hydrocodone Bitartrate  Hydrocodone Tannate  Hydromorphone Hydrochloride  Levorphanol Tartrate  Meperidine Hydrochloride  Methadone Hydrochloride  Morphine Sulfate  Oxycodone  Oxycodone Hydrochloride  Oxymorphone Hydrochloride  Tapentadol Hydrochloride |

**Table S5.** The list of ICD-10-CM codes related to relevant TEAEs

| **Adverse Event** | **Codes** |
| --- | --- |
| QT prolongation | I45.81 |
| Severe hypotension | I95.1, I95.2 |
| Atrial fibrillation | I48.0, I48.3, I48.4, I48.9, I48.91, I48.92 |
| Coronary artery disease, chest pain | I20, I20.0, I20.1, I20.2, I20.8, I20.9, I21, I21.0, I21.01, I21.02, I21.09, I21.1, I21.11, I21.19, I21.2, I21.21, I21.29, I21.3, I21.4, I21.9, I22, I22.0, I22.1, I22.2, I22.8, I22.9, I24.0, I24.8, I24.9 |
| Hypertension | I15, I15.8, I15.9, I16, I16.0, I16.1, I16.9 |
| Dizziness/vertigo | R42 |
| Somnolence | R40.0 |
| Confusion | R41.0, R41.4, R41.8, R41.82, R41.83, R41.84, R41.840, R41.841, R41.842, R41.843, R41.844, R41.89, R41.9 |
| Seizures | G40, G40.0, G40.00, G40.001, G40.009, G40.01, G40.011, G40.019, G40.1, G40.10, G40.101, G40.109, G40.11, G40.111, G40.119, G40.2, G40.20, G40.201, G40.209, G40.21, G40.211, G40.219, G40.3, G40.30, G40.301, G40.309, G40.31, G40.311, G40.319, G40.4, G40.40, G40.401, G40.409, G40.41, G40.411, G40.419, G40.5, G40.50, G40.501, G40.509, G40.8, G40.80, G40.801, G40.802, G40.803, G40.804, G40.89, G40.9, G40.90, G40.901, G40.909, G40.91, G40.911, G40.919 |
| Syncope | R55 |
| Cerebrovascular accident | I60, I60.0, I60.00, I60.01, I60.02, I60.1, I60.10, I60.11, I60.12, I60.2, I60.3, I60.30, I60.31, I60.32, I60.4, I60.5, I60.50, I60.51, I60.52, I60.6, I60.7, I60.8, I60.9, I61, I61.0, I61.1, I61.2, I61.3, I61.4, I61.5, I61.6, I61.8, I61.9, I62, I62.0, I62.00, I62.01, I62.02, I62.03, I62.1, I62.9, I63, I63.0, I63.00, I63.01, I63.011, I63.012, I63.013, I63.019, I63.02, I63.03, I63.031, I63.032, I63.033, I63.039, I63.09, I63.1, I63.10, I63.11, I63.111, I63.112, I63.113, I63.119, I63.12, I63.13, I63.131, I63.132, I63.133, I63.139, I63.19, I63.2, I63.20, I63.21, I63.211, I63.212, I63.213, I63.219, I63.22, I63.23, I63.231, I63.232, I63.233, I63.239, I63.29, I63.3, I63.30, I63.31, I63.311, I63.312, I63.313, I63.319, I63.32, I63.321, I63.322, I63.323, I63.329, I63.33, I63.331, I63.332, I63.333, I63.339, I63.34, I63.341, I63.342, I63.343, I63.349, I63.39, I63.4, I63.40, I63.41, I63.411, I63.412, I63.413, I63.419, I63.42, I63.421, I63.422, I63.423, I63.429, I63.43, I63.431, I63.432, I63.433, I63.439, I63.44, I63.441, I63.442, I63.443, I63.449, I63.49, I63.5, I63.50, I63.51, I63.511, I63.512, I63.513, I63.519, I63.52, I63.521, I63.522, I63.523, I63.529, I63.53, I63.531, I63.532, I63.533, I63.539, I63.54, I63.541, I63.542, I63.543, I63.549, I63.59, I63.6, I63.8, I63.81, I63.89, I63.9, I67, I67.0, I67.1, I67.2, I67.3, I67.4, I67.5, I67.6, I67.7, I67.8, I67.81, I67.82, I67.83, I67.84, I67.841, I67.848, I67.89, I67.9 |
| Nervousness | R45.0 |
| Visual disturbances | H53, H53.0, H53.00, H53.001, H53.002, H53.003, H53.009, H53.1, H53.10, H53.11, H53.12, H53.121, H53.122, H53.123, H53.129, H53.13, H53.131, H53.132, H53.133, H53.139, H53.14, H53.141, H53.142, H53.143, H53.149, H53.15, H53.16, H53.19, H53.2, H53.3, H53.30, H53.31, H53.32, H53.33, H53.34, H53.4, H53.40, H53.41, H53.411, H53.412, H53.413, H53.419, H53.42, H53.421, H53.422, H53.423, H53.429, H53.43, H53.431, H53.432, H53.433, H53.439, H53.45, H53.451, H53.452, H53.453, H53.459, H53.46, H53.461, H53.462, H53.469, H53.47, H53.48, H53.481, H53.482, H53.483, H53.489, H53.5, H53.50, H53.51, H53.52, H53.53, H53.54, H53.55, H53.59, H53.6, H53.60, H53.61, H53.62, H53.63, H53.69, H53.7, H53.71, H53.72, H53.8, H53.9 |
| Sleeplessness or insomnia | F519, F5102, F5109, F5101, F5103, F5109, F5119, F5111, F5112, F5119, F518, F513, G47419, G47411, G47429, G47421, G479, G4730, G4700, G4730, G4710, G4720, G478, G4730, F518, G478, Z72820 |
| Suicide ideation | R45.85, R45.850, R45.851, T14.91, T14.91XA, T14.91XD, T14.91XS, X71, X71.0, X71.0XXA, X71.0XXD, X71.0XXS, X71.1, X71.1XXA, X71.1XXD, X71.1XXS, X71.2, X71.2XXA, X71.2XXD, X71.2XXS, X71.3, X71.3XXA, X71.3XXD, X71.3XXS, X71.8, X71.8XXA, X71.8XXD, X71.8XXS, X71.9, X71.9XXA, X71.9XXD, X71.9XXS, X72, X72.XXXA, X72.XXXD, X72.XXXS, X73, X73.0, X73.0XXA, X73.0XXD, X73.0XXS, X73.1 In, X73.1XXA, X73.1XXD, X73.1XXS, X73.2 In, X73.2XXA, X73.2XXD, X73.2XXS, X73.8 In, X73.8XXA, X73.8XXD, X73.8XXS, X73.9 In, X73.9XXA, X73.9XXD, X73.9XXS, X74, X74.0, X74.01, X74.01XA, X74.01XD, X74.01XS, X74.02, X74.02XA, X74.02XD, X74.02XS, X74.09, X74.09XA, X74.09XD, X74.09XS, X74.8, X74.8XXA, X74.8XXD, X74.8XXS, X74.9, X74.9XXA, X74.9XXD, X74.9XXS, X75, X75.XXXA, X75.XXXD, X75.XXXS, X76, X76.XXXA, X76.XXXD, X76.XXXS, X77, X77.0, X77.0XXA, X77.0XXD, X77.0XXS, X77.1, X77.1XXA, X77.1XXD, X77.1XXS, X77.2, X77.2XXA, X77.2XXD, X77.2XXS, X77.3, X77.3XXA, X77.3XXD, X77.3XXS, X77.8, X77.8XXA, X77.8XXD, X77.8XXS, X77.9, X77.9XXA, X77.9XXD, X77.9XXS, X78, X78.0, X78.0XXA, X78.0XXD, X78.0XXS, X78.1, X78.1XXA, X78.1XXD, X78.1XXS, X78.2, X78.2XXA, X78.2XXD, X78.2XXS, X78.8, X78.8XXA, X78.8XXD, X78.8XXS, X78.9, X78.9XXA, X78.9XXD, X78.9XXS, X79, X79.XXXA, X79.XXXD, X79.XXXS, X80, X80.XXXA, X80.XXXD, X80.XXXS, X81, X81.0, X81.0XXA, X81.0XXD, X81.0XXS, X81.1, X81.1XXA, X81.1XXD, X81.1XXS, X81.8, X81.8XXA, X81.8XXD, X81.8XXS, X82, X82.0, X82.0XXA, X82.0XXD, X82.0XXS, X82.1, X82.1XXA, X82.1XXD, X82.1XXS, X82.2, X82.2XXA, X82.2XXD, X82.2XXS, X82.8, X82.8XXA, X82.8XXD, X82.8XXS, X83, X83.0, X83.0XXA, X83.0XXD, X83.0XXS, X83.1, X83.1XXA, X83.1XXD, X83.1XXS, X83.2, X83.2XXA, X83.2XXD, X83.2XXS, X83.8, X83.8XXA, X83.8XXD, X83.8XXS |
| Opioid abuse/dependence | F11, F11.1, F11.10, F11.11, F11.12, F11.120, F11.121, F11.122, F11.129, F11.13, F11.14, F11.15, F11.150, F11.151, F11.159, F11.18, F11.181, F11.182, F11.188, F11.19, F11.2, F11.20, F11.21, F11.22, F11.220, F11.221, F11.222, F11.229, F11.23, F11.24, F11.25, F11.250, F11.251, F11.259, F11.28, F11.281, F11.282, F11.288, F11.29, F11.9, F11.90, F11.91, F11.92, F11.920, F11.921, F11.922, F11.929, F11.93, F11.94, F11.95, F11.950, F11.951, F11.959, F11.98, F11.981, F11.982, F11.988, F11.99 |
| Opioid poisoning | T40, T40.0, T40.0X, T40.0X1, T40.0X1A, T40.0X1D, T40.0X1S, T40.0X2, T40.0X2A, T40.0X2D, T40.0X2S, T40.0X3, T40.0X3A, T40.0X3D, T40.0X3S, T40.0X4, T40.0X4A, T40.0X4D, T40.0X4S, T40.0X5, T40.0X5A, T40.0X5D, T40.0X5S, T40.0X6, T40.0X6A, T40.0X6D, T40.0X6S, T40.2, T40.2X, T40.2X1, T40.2X1A, T40.2X1D, T40.2X1S, T40.2X2, T40.2X2A, T40.2X2D, T40.2X2S, T40.2X3, T40.2X3A, T40.2X3D, T40.2X3S, T40.2X4, T40.2X4A, T40.2X4D, T40.2X4S, T40.2X5, T40.2X5A, T40.2X5D, T40.2X5S, T40.2X6, T40.2X6A, T40.2X6D, T40.2X6S, T40.3, T40.3X, T40.3X1, T40.3X1A, T40.3X1D, T40.3X1S, T40.3X2, T40.3X2A, T40.3X2D, T40.3X2S, T40.3X3, T40.3X3A, T40.3X3D, T40.3X3S, T40.3X4, T40.3X4A, T40.3X4D, T40.3X4S, T40.3X5, T40.3X5A, T40.3X5D, T40.3X5S, T40.3X6, T40.3X6A, T40.3X6D, T40.3X6S, T40.4, T40.41, T40.411, T40.411A, T40.411D, T40.411S, T40.412, T40.412A, T40.412D, T40.412S, T40.413, T40.413A, T40.413D, T40.413S, T40.414, T40.414A, T40.414D, T40.414S, T40.415, T40.415A, T40.415D, T40.415S, T40.416, T40.416A, T40.416D, T40.416S, T40.42, T40.421, T40.421A, T40.421D, T40.421S, T40.422, T40.422A, T40.422D, T40.422S, T40.423, T40.423A, T40.423D, T40.423S, T40.424, T40.424A, T40.424D, T40.424S, T40.425, T40.425A, T40.425D, T40.425S, T40.426, T40.426A, T40.426D, T40.426S, T40.49, T40.491, T40.491A, T40.491D, T40.491S, T40.492, T40.492A, T40.492D, T40.492S, T40.493, T40.493A, T40.493D, T40.493S, T40.494, T40.494A, T40.494D, T40.494S, T40.495, T40.495A, T40.495D, T40.495S, T40.496, T40.496A, T40.496D, T40.496S, T40.6, T40.60, T40.601, T40.601A, T40.601D, T40.601S, T40.602, T40.602A, T40.602D, T40.602S, T40.603, T40.603A, T40.603D, T40.603S, T40.604, T40.604A, T40.604D, T40.604S, T40.605, T40.605A, T40.605D, T40.605S, T40.606, T40.606A, T40.606D, T40.606S, T40.69, T40.691, T40.691A, T40.691D, T40.691S, T40.692, T40.692A, T40.692D, T40.692S, T40.693, T40.693A, T40.693D, T40.693S, T40.694, T40.694A, T40.694D, T40.694S, T40.695, T40.695A, T40.695D, T40.695S, T40.696, T40.696A, T40.696D, T40.696S |
| Headache | R51, R51.0, R51.9, G44.4, G44.40, G44.41 |
| Fatigue | R53, R53.1, R53.8, R53.81, R53.83 |
| Anaphylactic/allergic reactions | T78.2, T78.2XXA, T78.2XXD, T78.2XXS, T78.3, T78.3XXA, T78.3XXD, T78.3XXS, T78.4, T78.40, T78.40XA, T78.40XD, T78.40XS, T78.49, T78.49XA, T78.49XD, T78.49XS, T88.6, T88.6XXA, T88.6XXD, T88.6XXS, T88.7, T88.7XXA, T88.7XXD, T88.7XXS, T88.8, T88.8XXA, T88.8XXD, T88.8XXS, T88.9, T88.9XXA, T88.9XXD, T88.9XXS |
| Dehydration | E86, E86.0, E86.1, E86.9 |
| Dry mouth | R68.2 |
| Xerostomia | K11.7 |
| Sweating | R61 |
| Hot flushes | R23.2 |
| Sinusitis | J01, J01.0, J01.00, J01.01, J01.1, J01.10, J01.11, J01.2, J01.20, J01.21, J01.3, J01.30, J01.31, J01.4, J01.40, J01.41, J01.8, J01.80, J01.81, J01.9, J01.90, J01.91 |
| Nausea & Vomiting | R11, R11.0, R11.1, R11.10, R11.11, R11.12, R11.13, R11.14, R11.15, R11.2 |
| Constipation | K59.0, K59.00, K59.01, K59.02, K59.03, K59.09, K58.1 |
| Hepatotoxicity | K71, K71.0, K71.1, K71.10, K71.11, K71.2, K71.50, K71.51, K71.6, K71.7, K71.8, K71.9 |
| Cholecystitis | K81, K81.0, K81.2, K81.9 |
| Abdominal pain | R10, R10.0, R10.1, R10.10, R10.11, R10.12, R10.13, R10.2, R10.3, R10.30, R10.31, R10.32, R10.33, R10.8, R10.81, R10.811, R10.812, R10.813, R10.814, R10.815, R10.816, R10.817, R10.819, R10.82, R10.821, R10.822, R10.823, R10.824, R10.825, R10.826, R10.827, R10.829, R10.83, R10.84, R10.9 |
| Diarrhea | K59.1,  R19.7, K58.0, K52.1 |
| Loss of appetite, anorexia | F50,  F50.0,  F50.00,  F50.01,  F50.02,  F50.8,  F50.82,  F50.89,  F50.9, R63.0, R63.4, R63.6 |
| Adrenal insufficiency | E27.2, E27.3, E27.4, E27.40, E27.49 |
| Ankle fracture (risk of falls and fractures) | M84.3, M84.30, M84.30XA, M84.30XD, M84.30XG, M84.30XK, M84.30XP, M84.30XS, M84.31, M84.311, M84.311A, M84.311D, M84.311G, M84.311K, M84.311P, M84.311S, M84.312, M84.312A, M84.312D, M84.312G, M84.312K, M84.312P, M84.312S, M84.319, M84.319A, M84.319D, M84.319G, M84.319K, M84.319P, M84.319S, M84.32, M84.321, M84.321A, M84.321D, M84.321G, M84.321K, M84.321P, M84.321S, M84.322, M84.322A, M84.322D, M84.322G, M84.322K, M84.322P, M84.322S, M84.329, M84.329A, M84.329D, M84.329G, M84.329K, M84.329P, M84.329S, M84.33, M84.331, M84.331A, M84.331D, M84.331G, M84.331K, M84.331P, M84.331S, M84.332, M84.332A, M84.332D, M84.332G, M84.332K, M84.332P, M84.332S, M84.333, M84.333A, M84.333D, M84.333G, M84.333K, M84.333P, M84.333S, M84.334, M84.334A, M84.334D, M84.334G, M84.334K, M84.334P, M84.334S, M84.339, M84.339A, M84.339D, M84.339G, M84.339K, M84.339P, M84.339S, M84.34, M84.341, M84.341A, M84.341D, M84.341G, M84.341K, M84.341P, M84.341S, M84.342, M84.342A, M84.342D, M84.342G, M84.342K, M84.342P, M84.342S, M84.343, M84.343A, M84.343D, M84.343G, M84.343K, M84.343P, M84.343S, M84.344, M84.344A, M84.344D, M84.344G, M84.344K, M84.344P, M84.344S, M84.345, M84.345A, M84.345D, M84.345G, M84.345K, M84.345P, M84.345S, M84.346, M84.346A, M84.346D, M84.346G, M84.346K, M84.346P, M84.346S, M84.35, M84.350, M84.350A, M84.350D, M84.350G, M84.350K, M84.350P, M84.350S, M84.351, M84.351A, M84.351D, M84.351G, M84.351K, M84.351P, M84.351S, M84.352, M84.352A, M84.352D, M84.352G, M84.352K, M84.352P, M84.352S, M84.353, M84.353A, M84.353D, M84.353G, M84.353K, M84.353P, M84.353S, M84.359, M84.359A, M84.359D, M84.359G, M84.359K, M84.359P, M84.359S, M84.36, M84.361, M84.361A, M84.361D, M84.361G, M84.361K, M84.361P, M84.361S, M84.362, M84.362A, M84.362D, M84.362G, M84.362K, M84.362P, M84.362S, M84.363, M84.363A, M84.363D, M84.363G, M84.363K, M84.363P, M84.363S, M84.364, M84.364A, M84.364D, M84.364G, M84.364K, M84.364P, M84.364S, M84.369, M84.369A, M84.369D, M84.369G, M84.369K, M84.369P, M84.369S, M84.37, M84.371, M84.371A, M84.371D, M84.371G, M84.371K, M84.371P, M84.371S, M84.372, M84.372A, M84.372D, M84.372G, M84.372K, M84.372P, M84.372S, M84.373, M84.373A, M84.373D, M84.373G, M84.373K, M84.373P, M84.373S, M84.374, M84.374A, M84.374D, M84.374G, M84.374K, M84.374P, M84.374S, M84.375, M84.375A, M84.375D, M84.375G, M84.375K, M84.375P, M84.375S, M84.376, M84.376A, M84.376D, M84.376G, M84.376K, M84.376P, M84.376S, M84.377, M84.377A, M84.377D, M84.377G, M84.377K, M84.377P, M84.377S, M84.378, M84.378A, M84.378D, M84.378G, M84.378K, M84.378P, M84.378S, M84.379, M84.379A, M84.379D, M84.379G, M84.379K, M84.379P, M84.379S, M84.38, M84.38XA, M84.38XD, M84.38XG, M84.38XK, M84.38XP, M84.38XS, M84.4, M84.40, M84.40XA, M84.40XD, M84.40XG, M84.40XK, M84.40XP, M84.40XS, M84.41, M84.411, M84.411A, M84.411D, M84.411G, M84.411K, M84.411P, M84.411S, M84.412, M84.412A, M84.412D, M84.412G, M84.412K, M84.412P, M84.412S, M84.419, M84.419A, M84.419D, M84.419G, M84.419K, M84.419P, M84.419S, M84.42, M84.421, M84.421A, M84.421D, M84.421G, M84.421K, M84.421P, M84.421S, M84.422, M84.422A, M84.422D, M84.422G, M84.422K, M84.422P, M84.422S, M84.429, M84.429A, M84.429D, M84.429G, M84.429K, M84.429P, M84.429S, M84.43, M84.431, M84.431A, M84.431D, M84.431G, M84.431K, M84.431P, M84.431S, M84.432, M84.432A, M84.432D, M84.432G, M84.432K, M84.432P, M84.432S, M84.433, M84.433A, M84.433D, M84.433G, M84.433K, M84.433P, M84.433S, M84.434, M84.434A, M84.434D, M84.434G, M84.434K, M84.434P, M84.434S, M84.439, M84.439A, M84.439D, M84.439G, M84.439K, M84.439P, M84.439S, M84.44, M84.441, M84.441A, M84.441D, M84.441G, M84.441K, M84.441P, M84.441S, M84.442, M84.442A, M84.442D, M84.442G, M84.442K, M84.442P, M84.442S, M84.443, M84.443A, M84.443D, M84.443G, M84.443K, M84.443P, M84.443S, M84.444, M84.444A, M84.444D, M84.444G, M84.444K, M84.444P, M84.444S, M84.445, M84.445A, M84.445D, M84.445G, M84.445K, M84.445P, M84.445S, M84.446, M84.446A, M84.446D, M84.446G, M84.446K, M84.446P, M84.446S, M84.45, M84.451, M84.451A, M84.451D, M84.451G, M84.451K, M84.451P, M84.451S, M84.452, M84.452A, M84.452D, M84.452G, M84.452K, M84.452P, M84.452S, M84.453, M84.453A, M84.453D, M84.453G, M84.453K, M84.453P, M84.453S, M84.454, M84.454A, M84.454D, M84.454G, M84.454K, M84.454P, M84.454S, M84.459, M84.459A, M84.459D, M84.459G, M84.459K, M84.459P, M84.459S, M84.46, M84.461, M84.461A, M84.461D, M84.461G, M84.461K, M84.461P, M84.461S, M84.462, M84.462A, M84.462D, M84.462G, M84.462K, M84.462P, M84.462S, M84.463, M84.463A, M84.463D, M84.463G, M84.463K, M84.463P, M84.463S, M84.464, M84.464A, M84.464D, M84.464G, M84.464K, M84.464P, M84.464S, M84.469, M84.469A, M84.469D, M84.469G, M84.469K, M84.469P, M84.469S, M84.47, M84.471, M84.471A, M84.471D, M84.471G, M84.471K, M84.471P, M84.471S, M84.472, M84.472A, M84.472D, M84.472G, M84.472K, M84.472P, M84.472S, M84.473, M84.473A, M84.473D, M84.473G, M84.473K, M84.473P, M84.473S, M84.474, M84.474A, M84.474D, M84.474G, M84.474K, M84.474P, M84.474S, M84.475, M84.475A, M84.475D, M84.475G, M84.475K, M84.475P, M84.475S, M84.476, M84.476A, M84.476D, M84.476G, M84.476K, M84.476P, M84.476S, M84.477, M84.477A, M84.477D, M84.477G, M84.477K, M84.477P, M84.477S, M84.478, M84.478A, M84.478D, M84.478G, M84.478K, M84.478P, M84.478S, M84.479, M84.479A, M84.479D, M84.479G, M84.479K, M84.479P, M84.479S, M84.48, M84.48XA, M84.48XD, M84.48XG, M84.48XK, M84.48XP, M84.48XS, M84.6, M84.60, M84.60XA, M84.60XD, M84.60XG, M84.60XK, M84.60XP, M84.60XS, M84.61, M84.611, M84.611A, M84.611D, M84.611G, M84.611K, M84.611P, M84.611S, M84.612, M84.612A, M84.612D, M84.612G, M84.612K, M84.612P, M84.612S, M84.619, M84.619A, M84.619D, M84.619G, M84.619K, M84.619P, M84.619S, M84.62, M84.621, M84.621A, M84.621D, M84.621G, M84.621K, M84.621P, M84.621S, M84.622, M84.622A, M84.622D, M84.622G, M84.622K, M84.622P, M84.622S, M84.629, M84.629A, M84.629D, M84.629G, M84.629K, M84.629P, M84.629S, M84.63, M84.631, M84.631A, M84.631D, M84.631G, M84.631K, M84.631P, M84.631S, M84.632, M84.632A, M84.632D, M84.632G, M84.632K, M84.632P, M84.632S, M84.633, M84.633A, M84.633D, M84.633G, M84.633K, M84.633P, M84.633S, M84.634, M84.634A, M84.634D, M84.634G, M84.634K, M84.634P, M84.634S, M84.639, M84.639A, M84.639D, M84.639G, M84.639K, M84.639P, M84.639S, M84.64, M84.641, M84.641A, M84.641D, M84.641G, M84.641K, M84.641P, M84.641S, M84.642, M84.642A, M84.642D, M84.642G, M84.642K, M84.642P, M84.642S, M84.649, M84.649A, M84.649D, M84.649G, M84.649K, M84.649P, M84.649S, M84.65, M84.650, M84.650A, M84.650D, M84.650G, M84.650K, M84.650P, M84.650S, M84.651, M84.651A, M84.651D, M84.651G, M84.651K, M84.651P, M84.651S, M84.652, M84.652A, M84.652D, M84.652G, M84.652K, M84.652P, M84.652S, M84.653, M84.653A, M84.653D, M84.653G, M84.653K, M84.653P, M84.653S, M84.659, M84.659A, M84.659D, M84.659G, M84.659K, M84.659P, M84.659S, M84.66, M84.661, M84.661A, M84.661D, M84.661G, M84.661K, M84.661P, M84.661S, M84.662, M84.662A, M84.662D, M84.662G, M84.662K, M84.662P, M84.662S, M84.663, M84.663A, M84.663D, M84.663G, M84.663K, M84.663P, M84.663S, M84.664, M84.664A, M84.664D, M84.664G, M84.664K, M84.664P, M84.664S, M84.669, M84.669A, M84.669D, M84.669G, M84.669K, M84.669P, M84.669S, M84.67, M84.671, M84.671A, M84.671D, M84.671G, M84.671K, M84.671P, M84.671S, M84.672, M84.672A, M84.672D, M84.672G, M84.672K, M84.672P, M84.672S, M84.673, M84.673A, M84.673D, M84.673G, M84.673K, M84.673P, M84.673S, M84.674, M84.674A, M84.674D, M84.674G, M84.674K, M84.674P, M84.674S, M84.675, M84.675A, M84.675D, M84.675G, M84.675K, M84.675P, M84.675S, M84.676, M84.676A, M84.676D, M84.676G, M84.676K, M84.676P, M84.676S, M84.68, M84.68XA, M84.68XD, M84.68XG, M84.68XK, M84.68XP, M84.68XS, M84.7, M84.75, M84.750, M84.750A, M84.750D, M84.750G, M84.750K, M84.750P, M84.750S, M84.751, M84.751A, M84.751D, M84.751G, M84.751K, M84.751P, M84.751S, M84.752, M84.752A, M84.752D, M84.752G, M84.752K, M84.752P, M84.752S, M84.753, M84.753A, M84.753D, M84.753G, M84.753K, M84.753P, M84.753S, M84.754, M84.754A, M84.754D, M84.754G, M84.754K, M84.754P, M84.754S, M84.755, M84.755A, M84.755D, M84.755G, M84.755K, M84.755P, M84.755S, M84.756, M84.756A, M84.756D, M84.756G, M84.756K, M84.756P, M84.756S, M84.757, M84.757A, M84.757D, M84.757G, M84.757K, M84.757P, M84.757S, M84.758, M84.758A, M84.758D, M84.758G, M84.758K, M84.758P, M84.758S, M84.759, M84.759A, M84.759D, M84.759G, M84.759K, M84.759P, M84.759S |
| Osteoarthritis | M15.3, M15.8, M15.9, M16.6, M16.7, M16.9, M17.4, M17.5, M17.9, M18.4, M18.5, M18.50, M18.51, M18.52, M18.9, M19.2, M19.21, M19.211, M19.212, M19.219, M19.22, M19.221, M19.222, M19.229, M19.23, M19.231, M19.232, M19.239, M19.24, M19.241, M19.242, M19.249, M19.27, M19.271, M19.272, M19.279, M19.29, M19.9, M19.90, M19.93, |
| Respiratory depression | J96.0, J96.00, J96.01, J96.02, J96.9, J96.90, J96.91, J96.92, R09.2 |
| Pneumonia | J17, J18, J18.0, J18.1, J18.2, J18.8, J18.9, A37.91, J84.11, J84.111, J84.112, J84.113, J84.114, J84.115, J84.116, J84.117 |
| Cellulitis | L03.01, L03.011, L03.012, L03.019, L03.03, L03.031, L03.032, L03.039, L03.1, L03.11, L03.111, L03.112, L03.113, L03.114, L03.115, L03.116, L03.119, L03.2, L03.21, L03.211, L03.213, L03.22, L03.221, L03.3, L03.31, L03.311, L03.312, L03.313, L03.314, L03.315, L03.316, L03.317, L03.319, L03.8, L03.81, L03.811, L03.818, L03.9, L03.90, H60.1, H60.10, H60.11, H60.12, H60.13, H05.01, H05.011, H05.012, H05.013, H05.019, N73.0, K12.2, N48.22 |
| Pruritus | L29, L29.0, L29.1, L29.2, L29.3, L29.8, L29.9 |
| Erythema | L51, L51.0, L51.1, L51.2, L51.3, L51.8, L51.9, L52, L53, L53.0, L53.1, L53.2, L53.3, L53.8, L53.9, L54 |
| Rash | R21, D72.12 |
| Irritation | L24, L24.4, L24.8, L24.89, L24.9 |
| Micturition difficulty, urinary retention | R30, R30.0, R30.1, R30.9, R32, R33, R33.0, R33.8, R33.9, R34, R39, R39.1, R39.11, R39.12, R39.13, R39.14, R39.15, R39.16, R39.19, R39.191, R39.192, R39.198, R39.8, R39.81, R39.89, R39.9 |

**Table S6.** Demographic characteristics of matched patients in analysis #1

|  | **Belbuca®** **(N=341)** | **CII Opioids (N=1,321)** | **P-value*** |
| --- | --- | --- | --- |
| Age, mean (SD) | 49.3 (10.0) | 48.9 (11.3) | 0.517 |
| **Gender, n (%)** |  |  |  |
| Male | 115 (33.7) | 421 (31.9) | 0.514 |
| Female | 226 (66.3) | 900 (68.1) | 0.514 |
| **Health Plan, n (%)** |  |  |  |
| Basic/Major Medical | 0 (0.0) | 0 (0.0) | - |
| Comprehensive | 11 (3.2) | 45 (3.4) | 0.869 |
| Exclusive Provider Organization | 4 (1.2) | 11 (0.8) | 0.525 |
| Health Maintenance Organization | 40 (11.7) | 171 (12.9) | 0.548 |
| Non-Capitated Point-of-Service | 26 (7.6) | 100 (7.6) | 0.973 |
| POS with Capitation | 1 (0.3) | 2 (0.2) | 0.498 |
| Preferred Provider Organization | 162 (47.5) | 633 (47.9) | 0.892 |
| Consumer-Driven Health Plan | 59 (17.3) | 219 (16.6) | 0.750 |
| High-Deductible Health Plan | 35 (10.3) | 123 (9.3) | 0.593 |
| Unknown | 3 (0.9) | 17 (1.3) | 0.781 |
| **Region, n (%)** |  |  |  |
| North-East | 25 (7.3) | 98 (7.4) | 0.956 |
| North-Central | 46 (13.5) | 183 (13.9) | 0.862 |
| South | 235 (68.9) | 908 (68.7) | 0.949 |
| West | 34 (10.0) | 126 (9.5) | 0.809 |
| Unknown | 1 (0.3) | 6 (0.5) | 0.682 |

**Chi-square test was performed for categorical variables and an independent T-test for continuous variables*

**Table S7.** Clinical characteristics of matched patients in analysis #1

|  | **Belbuca® (N=341)** | **CII Opioids (N=1,321)** | **P-value*** |
| --- | --- | --- | --- |
| **Charlson Comorbidity Index** |  |  |  |
| 0 | 189 (55.4) | 756 (57.2) | 0.549 |
| 1 | 72 (21.1) | 290 (22.0) | 0.738 |
| 2 | 22 (6.5) | 75 (5.7) | 0.587 |
| 3 | 33 (9.7) | 115 (8.7) | 0.574 |
| 4+ | 25 (7.3) | 85 (6.4) | 0.553 |
| Charlson Comorbidity Index, mean (SD) | 1.0 (1.6) | 0.9 (1.5) | 0.407 |
| **Charlson Comorbidity Index Components** | | | |
| Myocardial infarction | 2 (0.6) | 6 (0.5) | 0.671 |
| Congestive heart failure | 9 (2.6) | 23 (1.7) | 0.282 |
| Peripheral vascular disease | 9 (2.6) | 28 (2.1) | 0.562 |
| Cerebrovascular disease | 13 (3.8) | 39 (3.0) | 0.416 |
| Dementia | 0 (0.0) | 0 (0.0) | - |
| Chronic pulmonary disease | 47 (13.8) | 170 (12.9) | 0.655 |
| Rheumatic disease | 20 (5.9) | 57 (4.3) | 0.225 |
| Peptic ulcer disease | 5 (1.5) | 18 (1.4) | 0.799 |
| Mild liver disease | 15 (4.4) | 55 (4.2) | 0.847 |
| Moderate or severe liver disease | 2 (0.6) | 6 (0.5) | 0.671 |
| Diabetes without chronic complications | 52 (15.2) | 244 (18.5) | 0.166 |
| Diabetes with chronic complications | 49 (14.4) | 163 (12.3) | 0.316 |
| Hemiplegia or paraplegia | 2 (0.6) | 6 (0.5) | 0.671 |
| Renal disease | 10 (2.9) | 46 (3.5) | 0.616 |
| Malignancy | 9 (2.6) | 35 (2.6) | 0.992 |
| Metastatic solid tumor | 2 (0.6) | 4 (0.3) | 0.609 |
| AIDS/HIV | 2 (0.6) | 7 (0.5) | 1.000 |
| **Mental Disorders** |  |  |  |
| Anxiety | 102 (29.9) | 385 (29.1) | 0.781 |
| Bipolar disorder | 17 (5.0) | 59 (4.5) | 0.683 |
| Depression | 93 (27.3) | 343 (26.0) | 0.625 |
| Sleep disorder | 40 (11.7) | 145 (11.0) | 0.693 |
| Psychosis | 10 (2.9) | 35 (2.6) | 0.774 |
| Post-traumatic stress syndrome | 9 (2.6) | 39 (3.0) | 0.758 |
| **Chronic Pain-Specific Comorbidities** |  |  |  |
| Joint pain | 158 (46.3) | 618 (46.8) | 0.882 |
| Musculoskeletal disorders | 274 (80.4) | 1067 (80.8) | 0.861 |
| Diabetic neuropathy | 16 (4.7) | 53 (4.0) | 0.575 |
| Other neuropathies | 106 (31.1) | 408 (30.9) | 0.943 |
| Spine disorders | 236 (69.2) | 916 (69.3) | 0.962 |
| Fibromyalgia | 63 (18.5) | 230 (17.4) | 0.646 |
| **Other Comorbidities** |  |  |  |
| COVID infection | 18 (5.3) | 59 (4.5) | 0.525 |

**Chi-square test was performed for categorical variables and an independent T-test for continuous variables*

**Table S8.** Demographic characteristics of matched patients in the analysis #2

|  | **Belbuca® (N=321)** | **Bup. Patch (N=321)** | **P-value*** |
| --- | --- | --- | --- |
| Age, mean (SD) | 49.4 (9.9) | 50.5 (10.0) | 0.139 |
| **Gender, n (%)** |  |  |  |
| Male | 104 (32.4) | 109 (34.0) | 0.675 |
| Female | 217 (67.6) | 212 (66.0) | 0.675 |
| **Health Plan, n (%)** |  |  |  |
| Basic/Major Medical | 0 (0.0) | 0 (0.0) | - |
| Comprehensive | 14 (4.4) | 16 (5.0) | 0.708 |
| Exclusive Provider Organization | 5 (1.6) | 4 (1.2) | 1.000 |
| Health Maintenance Organization | 37 (11.5) | 45 (14.0) | 0.344 |
| Non-Capitated Point-of-Service | 26 (8.1) | 25 (7.8) | 0.884 |
| POS with Capitation | 1 (0.3) | 0 (0.0) | 1.000 |
| Preferred Provider Organization | 165 (51.4) | 164 (51.1) | 0.937 |
| Consumer-Driven Health Plan | 35 (10.9) | 35 (10.9) | 1.000 |
| High-Deductible Health Plan | 35 (10.9) | 29 (9.0) | 0.429 |
| Unknown | 3 (0.9) | 3 (0.9) | 1.000 |
| **Region, n (%)** |  |  |  |
| North-East | 25 (7.8) | 21 (6.5) | 0.540 |
| North-Central | 43 (13.4) | 43 (13.4) | 1.000 |
| South | 225 (70.1) | 225 (70.1) | 1.000 |
| West | 27 (8.4) | 32 (10.0) | 0.495 |
| Unknown | 1 (0.3) | 0 (0.0) | 1.000 |

**Chi-square test was performed for categorical variables and an independent T-test for continuous variables*

**Table S9.** Clinical characteristics of matched patients in the analysis #2

|  | **Belbuca® (N=321)** | **Bup. Patch (N=321)** | **P-value*** |
| --- | --- | --- | --- |
| **Charlson Comorbidity Index** |  |  |  |
| 0 | 172 (53.6) | 178 (55.5) | 0.634 |
| 1 | 66 (20.6) | 63 (19.6) | 0.768 |
| 2 | 23 (7.2) | 23 (7.2) | 1.000 |
| 3 | 32 (10.0) | 20 (6.2) | 0.083 |
| 4+ | 28 (8.7) | 37 (11.5) | 0.239 |
| Charlson Comorbidity Index, mean (SD) | 1.1 (1.6) | 1.2 (1.9) | 0.605 |
| **Charlson Comorbidity Index Components** | | | |
| Myocardial infarction | 2 (0.6) | 0 (0.0) | 0.499 |
| Congestive heart failure | 8 (2.5) | 4 (1.2) | 0.383 |
| Peripheral vascular disease | 8 (2.5) | 5 (1.6) | 0.577 |
| Cerebrovascular disease | 12 (3.7) | 6 (1.9) | 0.151 |
| Dementia | 0 (0.0) | 1 (0.3) | 1.000 |
| Chronic pulmonary disease | 50 (15.6) | 42 (13.1) | 0.368 |
| Rheumatic disease | 18 (5.6) | 29 (9.0) | 0.096 |
| Peptic ulcer disease | 6 (1.9) | 5 (1.6) | 1.000 |
| Mild liver disease | 14 (4.4) | 24 (7.5) | 0.094 |
| Moderate or severe liver disease | 4 (1.2) | 3 (0.9) | 1.000 |
| Diabetes without chronic complications | 54 (16.8) | 54 (16.8) | 1.000 |
| Diabetes with chronic complications | 51 (15.9) | 50 (15.6) | 0.914 |
| Hemiplegia or paraplegia | 3 (0.9) | 0 (0.0) | 0.249 |
| Renal disease | 11 (3.4) | 14 (4.4) | 0.541 |
| Malignancy | 8 (2.5) | 14 (4.4) | 0.193 |
| Metastatic solid tumor | 1 (0.3) | 5 (1.6) | 0.217 |
| AIDS/HIV | 2 (0.6) | 1 (0.3) | 1.000 |
| **Mental Disorders** |  |  |  |
| Anxiety | 109 (34.0) | 109 (34.0) | 1.000 |
| Bipolar disorder | 21 (6.5) | 13 (4.0) | 0.159 |
| Depression | 99 (30.8) | 84 (26.2) | 0.190 |
| Sleep disorder | 44 (13.7) | 50 (15.6) | 0.503 |
| Psychosis | 12 (3.7) | 8 (2.5) | 0.364 |
| Post-traumatic stress syndrome | 13 (4.0) | 24 (7.5) | 0.062 |
| **Chronic Pain-Specific Comorbidities** |  |  |  |
| Joint pain | 151 (47.0) | 169 (52.6) | 0.155 |
| Musculoskeletal disorders | 260 (81.0) | 255 (79.4) | 0.620 |
| Diabetic neuropathy | 15 (4.7) | 13 (4.0) | 0.699 |
| Other neuropathies | 107 (33.3) | 119 (37.1) | 0.321 |
| Spine disorders | 227 (70.7) | 230 (71.7) | 0.794 |
| Fibromyalgia | 64 (19.9) | 83 (25.9) | 0.074 |
| **Other Comorbidities** |  |  |  |
| COVID infection | 18 (5.6) | 12 (3.7) | 0.262 |

**Chi-square test was performed for categorical variables and an independent T-test for continuous variables*
